# Promoting Comprehensive Care for People With Rare Diseases in a Tertiary Care Setting in Brazil: Protocol for a Mixed Methods Implementation Study

**Published:** 2025-08-18

**Authors:** Domingos Alves, Filipe Andrade Bernardi, Vinicius Costa Lima, Diego Bettiol Yamada, Tatiana Takahasi Komoto, Michele de Souza Seixas, Victor Cassão, Leticia Fontanelli Straube de Souza, Amaury Lelis Dal Fabbro, Têmis Maria Félix, Ricardo Cavalli, Victor Evangelista de Faria Ferraz

**Affiliations:** 1 Department of Social Medicine, Ribeirão Preto Medical School, University of São Paulo Ribeirão Preto Brazil; 2 Ribeirão Preto Medical School, University of São Paulo, Ribeirão Preto, Brazil Ribeirão Preto Brazil; 3 RISE – Health Research Network (Rede de Investigação em Saúde), Department of Community Medicine, Information and Health Decision-Making Faculty of Medicine Universidade do Porto Porto Portugal; 4 Institute for Systems and Computers Engineering at Coimbra Institute for Systems Engineering and Computers Coimbra Portugal; 5 Hospital de Clínicas de Porto Alegre Porto Alegre Brazil; 6 Clinical Hospital of the Faculty of Medicine of Ribeirão Preto University of São Paulo-USP Ribeirão Preto Brazil

**Keywords:** rare disease, digital health, comprehensive care, public health policy, implementation science

## Abstract

**Background:**

Rare diseases (RDs) have gained attention in public policy due to their complexity and low prevalence and the challenges they present in health care settings. Comprehensive care for people with RDs requires strengthening of services, programs, and care levels. It is imperative to make digital health tools available to support epidemiological surveillance, facilitate patient follow-up, and enhance the education and awareness of health care professionals (HCPs) regarding these conditions.

**Objective:**

This study aims to implement enhanced actions aligned with the Brazilian National Policy for Comprehensive Care for People with Rare Diseases to improve the attention and care provided to individuals. This will be achieved by developing a computational tool and establishing guidelines to optimize the regulation of the reference services for RDs, providing updated information to expedite diagnosis and promote collaboration.

**Methods:**

The methodology includes mapping the regulation processes in the Clinics Hospital of Ribeirão Preto Health Complex. Participants will include HCPs from the hospital complex and associated primary care centers and patients with a confirmed or suspected RD selected through medical records and patient associations. Data collection methods include questionnaires, semistructured interviews, and patient tracking using health information systems. The project will apply the 5W2H (what, who, where, when, why, how, and how much) framework to align tasks, responsibilities, and resources. Integrating digital tools adhering to the findability, accessibility, interoperability, and reusability principles will promote real-time monitoring, improved resource allocation, and enhanced workflow efficiency. Training initiatives will boost awareness and capacity among HCPs, while the computational tool seeks to streamline patient follow-up and tracking. Digital integration and interoperability will reduce referral process inefficiencies and support evidence-based decision-making. Education and awareness campaigns will fill knowledge gaps among HCPs and patients.

**Results:**

Ethics approval was granted on December 9, 2024. We conducted technical meetings with the IT team to align procedures for obtaining secondary data. Concurrently, we identified key guidelines and applied a knowledge management maturity questionnaire based on which we began mapping RD-related management processes. A computational ontology is currently under development to ensure semantic interoperability. This framework supported initiatives promoting education and awareness regarding RDs, targeting HCPs and patients.

**Conclusions:**

The study emphasizes the potential of digital health solutions to transform RD care by improving coordination, resource allocation, and stakeholder education. When effectively adopted, these solutions can enhance access to specialized care, reduce inefficiencies, and inform public health policies. Future efforts will focus on expanding the tool’s implementation, refining its functionalities, and evaluating its long-term impact on patient outcomes and system efficiency. This initiative highlights the crucial role of integrating digital technologies in managing RDs and underscores the need for ongoing collaboration among health care stakeholders to achieve sustainable improvements in patient care and policy development.

**International Registered Report Identifier (IRRID):**

PRR1-10.2196/68949

## Introduction

### Background: Evolution of Public Policies for Rare Diseases

Attention to rare diseases (RDs) has increasingly gained relevance in public policy discussions. The National Policy for Comprehensive Care for People with Rare Diseases (NPCCPRD), established by Ordinance 199/2014 of the Brazilian Ministry of Health (MoH), aims to reduce mortality, morbidity, and secondary manifestations while improving the quality of life and equitable access to health services [1].

The policy’s main objectives include strengthening the RD care network, enhancing the qualifications of health care professionals (HCPs), promoting scientific research, and encouraging knowledge production. Furthermore, the ordinance emphasizes coordination between institutions and governmental bodies, improving information flow through more robust registration and surveillance systems and increasing education and awareness about RDs [1]. However, implementing these policies faces significant challenges and difficulties [2].

Although RDs collectively affect a large portion of the global population, individual conditions remain rare. These diseases are characterized by their complexity, diversity, and severity, often presenting debilitating and chronic symptoms. It is estimated that there are >7000 identified RDs [3], making it challenging to establish standardized approaches and universal guidelines applicable to all RDs. The need for comprehensive, updated information on incidence and prevalence also hampers the planning of health services and the adequate allocation of resources [4].

A significant obstacle to progress is low awareness and knowledge among HCPs and society. The lack of familiarity and specialized training can delay early diagnosis and appropriate referral of patients with RDs [5]. Therefore, coordination and integration between various sectors, including health care, research, the pharmaceutical industry, and patient organizations, are crucial for the effective development and implementation of RD initiatives [4]. Collaboration between these actors is often complex, given divergent interests, resource limitations, and coordination challenges. However, promoting partnerships between government institutions, patient organizations, HCPs, and the academic sector can significantly improve patients’ quality of life [5].

Over time, the evolution of public policy for RDs has been a gradual but continuous process. Initially, these conditions were ignored by government authorities and the pharmaceutical industry. However, social movements and patient organizations worldwide have voiced the needs of individuals with RDs, contributing to the recognition of RDs as a global public health issue [6]. Advances in genetics and medicine have facilitated a greater understanding of RDs and more accurate diagnoses. This progress has led to identifying patient groups, creating research networks, and establishing reference centers for RDs (RCRDs) for high-quality care [2]. Since the publication of the NPCCPRD, 27 RCRDs have been accredited by the MoH, reflecting progress in policy implementation and improving care for people with RDs in Brazil. However, challenges remain, such as expanding coverage and ensuring adequate resources for comprehensive care for these patients. Continuous monitoring and improvement of the NPCCPRD are essential to address these challenges and promote more effective and inclusive care [7].

Discussions on public policies to support the population with RD have intensified recently. In addition to the NPCCPRD, São Paulo State Law 14.806 emphasizes raising awareness about these diseases [8]. Furthermore, São Paulo State Law 15.669 of January 12, 2015, addresses the RD treatment policy in São Paulo and outlines the objectives of the RCRD, which include diagnosing and mapping RDs and promoting effective RD treatment, monitoring patients, serving as research and teaching centers, and providing early diagnosis to minimize additional disabilities [9]. Globally, the inclusion of RDs in the World Health Assembly agenda in 2019 was a landmark, establishing RDs as a priority in international health policy [10].

The Council of Rare Diseases International highlighted the potential of digital health technology in accelerating access to diagnosis and specialized care for patients with RDs. The need for standardized definitions, classifications, codifications, and validations by the World Health Organization, including tools such as the Human Phenotype Ontology and the *International Classification of Diseases*, was emphasized as the key to success [11]. The European Reference Networks demonstrate how digital technologies can improve care coordination, facilitate expert collaboration, and accelerate research, promoting a patient-centered digital transformation [12,13].

As we advance into the digital era, new obstacles and opportunities arise in the evolution of public policies. Comprehensive care for people with RDs requires not only the implementation of basic actions for diagnosis and care but also the strengthening of existing services, programs, and levels of care. These efforts depend on the health care network’s financial, technical, human, and infrastructural resources [14,15]. Therefore, it is crucial to continuously evaluate and improve the policy landscape for RDs, adapting to new realities and ensuring that patients receive the necessary support to face the challenges posed by their conditions.

### Objectives

In this scenario, developing digital health tools becomes imperative to contribute to epidemiological surveillance, patient follow-up, and awareness among HCPs. These tools should facilitate patient registration, monitoring, and follow-up as well as store information related to treatments, tests, and hospitalizations. Digital tools can significantly improve data collection, reduce losses, and allow better analysis and management of knowledge, ultimately leading to better care and disease management.

Thus, the primary objective of this paper is to implement and enhance actions aligned with the NPCCPRD to improve the attention and care provided to individuals with RDs. This will be achieved by developing a computational tool and establishing guidelines to optimize the regulation (referral and counterreferral) for the reference services for RDs (RSRDs). This includes mapping the regulation scenarios for patients within the Clinics Hospital of Ribeirão Preto (HCRP [Hospital das Clínicas de Ribeirão Preto]) care network. In addition, continuous evaluation and refinement of the digital health strategies applied within the HCRP complex are needed to expand the reach, effectiveness, adoption, implementation, and maintenance of actions as per the NPCCPRD. Finally, awareness programs and specialized second opinion tools should be implemented to support continuous education, informed decision-making, and the discussion on confirmed or suspected RD cases within the care network.

Given the importance of records for conducting scientific research that can promote the evaluation and monitoring of patients and reduce diagnosis time through a national database, the project aims to provide information on symptoms, diagnostic tests, and available RSRDs to those interested in the subject, similar to the approaches described by Gong et al [16] and Vandeborne et al [17]. The absence of public policies directed at RDs [7] intensifies the urgency of addressing this issue.

## Methods

### Study Design

This applied research with a mixed methods approach will follow procedures based on action research, literature review, surveys, and field research. Action research will foster change through continuous collaboration with HCPs and other stakeholders to address specific issues related to RD care. The literature review will provide a comprehensive understanding of existing knowledge and gaps in the field, guiding the development of new insights and approaches. Surveys will be conducted to collect quantitative data from a broad range of participants, ensuring a representative understanding of the current state and challenges of RD care. Field research will involve in-depth qualitative studies, including interviews and observations, to gather detailed contextual information and firsthand accounts of experiences within the health care network. The interview guide is provided in Multimedia Appendix 1. This combined methodology aims to integrate diverse perspectives and data sources, enhancing the robustness and applicability of the research findings [18].

To structure the knowledge consolidated during the initial phases of the study, a computational ontology will be developed, focused on the domain of RDs within the context of the Unified Health System (SUS; Sistema Único de Saúde). This development will follow the guidelines established by the World Wide Web Consortium for the standardization of informational elements on the web. The ontology will serve as the foundation for designing an interoperable informational repository structure, an essential aspect for optimizing communication within a networked health care system [19,20].

Subsequently, to gain a deeper understanding of the management processes intrinsic to the care of patients with RDs at the study site, these processes will be mapped using the Business Process Model and Notation. This is a well-established approach aimed at accurately and transparently modeling organizational processes, enabling the clear identification of opportunities for improvement and strategic points for generating service performance indicators. In addition to supporting decision-making and achieving better management outcomes, this methodology contributes to enhancing the quality of services provided and holds potential for reducing operational costs within the organization [21,22].

The 5W2H (what, who, where, when, why, how, and how much) methodology, a widely used tool in project management, was used to structure and organize the proposed actions. This approach is based on addressing 7 fundamental questions: “what” (what will be done?), “why” (why will it be done?), “who” (who will be responsible?), “when” (when will it be done?), ‘where’ (where will it be executed?), “how” (how will it be done?), and “how much” (how much will it cost?). This methodology provides a clear, actionable framework for defining tasks, responsibilities, and resources, ensuring stakeholder efficiency and alignment [23]. Adopting structured frameworks in health information management is essential to optimize decision-making and improve service quality, particularly in resource-constrained environments such as the RD scenarios. This ensures a comprehensive understanding of the project’s scope and facilitates the elimination of inefficiencies, as recommended in lean management principles applied to health care systems [24].

### Study Participants

Certified as an RSRD in 2019, the HCRP has had a medical genetics service since 1974 and is a reference center in RD care [25]. The hospital offers high-complexity clinical and laboratory investigations, including genetic counseling and genomic testing [26]. The use of this system in health services to integrate follow-up actions and the resolution of these actions will be evaluated. In addition, a sociotechnical approach will be applied for training, necessary system modifications and awareness and education actions that will be implemented for primary care centers and HCRP units.

Primary care centers were created to support didactic activities in primary and secondary care, promoting interinstitutional integration and the technical-scientific improvement of faculty. Furthermore, they aim to train HCPs, develop human resources, and produce relevant scientific knowledge. Therefore, the impact of implementing integration and interoperability methods on data quality improvement and organizational management will also be assessed.

The research will use diverse criteria to select participants, including geographic diversity and representation of different RD groups. A comprehensive mapping of patients, HCPs, and health managers in specific health services units will be conducted using medical records, patient associations, information systems, and care networks. To collect data, questionnaires and semistructured interviews will be adapted to address aspects of access to the care network concerning RDs. The type of registry to be implemented will be a notification system, allowing for the systematic collection of information on RD cases. The development process of the registry will include the definition of indicators and the evaluation of data quality obtained according to current legislation and the region’s specific needs [27,28].

The study participants comprise 2 main groups: HCPs working within the network of the HCRP and the broader health care network of Ribeirão Preto and patients with medical records in associated databases. Eligibility criteria for HCPs include the type of institution where they work, such as basic health units and family health centers, excluding those who do not directly manage patients with RDs. For patients, inclusion criteria include a confirmed or suspected RD, relevant signs and symptoms, and being under follow-up at the HCRP. Patients with incomplete or low-quality records will be excluded. Patient data will be accessed through the HCRP health information systems and municipal health records, with some participants potentially excluded if they do not meet the established criteria or if their records lack sufficient information.

**Figure 1:**
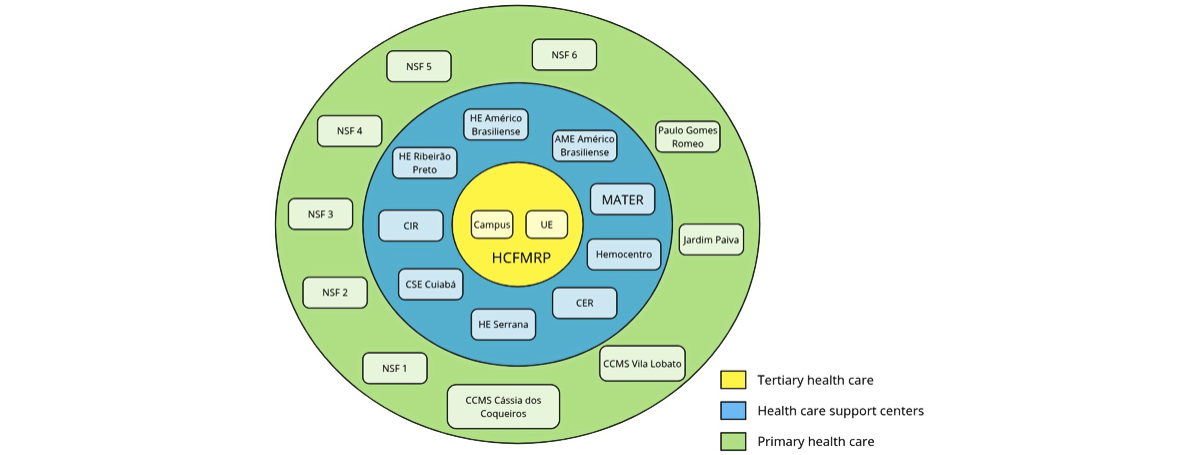
Clinics Hospital of Ribeirão Preto (Hospital das Clínicas de Ribeirão Preto) unit composition. AME: specialty medical outpatient clinic; CCMS: Community Health and Social Services Center; CER: rehabilitation center; CIR: integrated rehabilitation center; CSE: teaching health center; HE: state hospital; NSF: family health units; UE: emergency hospital.

However, the HCRP Academic Health Complex and its various units and services, as presented in Figure 1, face challenges in regulation, the implementation of procedures, and the financial and epidemiological aspects of RDs. Financial difficulties include underfunding and a need for more resources for tests and procedures, which, in turn, impact the hospital’s ability to provide quality services. Implementing methods is also challenging due to unclear regulations and difficulties in internal agreements. The lack of knowledge about the epidemiology of RDs is another barrier, making it difficult to plan and implement appropriate health policies for these conditions.

### Phases

**Figure 2:**
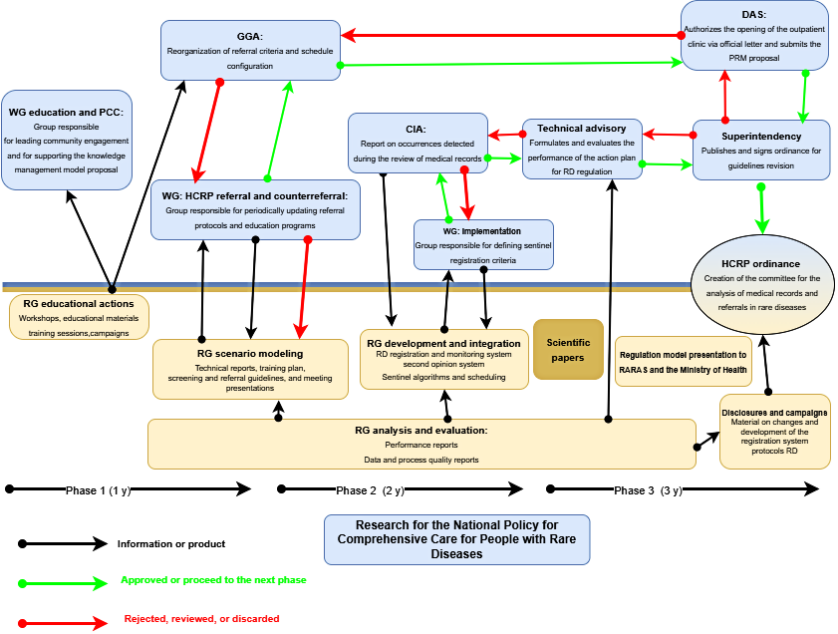
Alignment between the scientific and public management components. CIA: Information and Informatics Center; DAS: department of health care; HCRP: Clinics Hospital of Ribeirão Preto (Hospital das Clínicas de Ribeirão Preto); GGA: general outpatient management; PCC: primary care center; RD: rare disease; RG: research group; WG: working group.

Although the project’s 3 phases are articulated, as shown in Figure 2, the flowchart provides a degree of independence between its stages. In phase 1, there is a scenario of detailed diagnosis and planning of the needs and challenges faced by HCRP in managing people with RDs within its care network. A survey of current practices in registration, monitoring, and referral will be conducted, identifying points for improvement to support the definition of protocols and a referral and counterreferral model to be implemented. Phase 2 corresponds to implementing and evaluating the planned strategies and interventions that will be developed to improve these patients’ registration, monitoring, and referral.

Finally, in phase 3, the impact of the results obtained throughout the study will be analyzed from 2 main aspects: the availability of the achieved results about public management needs and the impacts generated by the research on the public management process during the project’s term. It is important to emphasize that the 3 action lines specified earlier are transversal to the project, which is synergistically presented in the 3 phases mentioned earlier.

### Computational Infrastructure

The data collection instruments will use consolidated and validated international standards, such as the *International Classification of Diseases, 10th Revision*, Human Phenotype Ontology, ORPHAcode, and OMIM, enabling comparison with data from other platforms, such as Orphanet [14,29,30]. These instruments were established according to the NPCCPRD guidelines and World Health Organization recommendations and validated by the National Rare Diseases Network (RARAS; Rede Nacional de Doenças Raras) experts. The selection of registry variables will be based on a systematic review conducted by the RARAS group, which verified the RD minimum dataset used in health networks in different countries [12].

REDCap (Research Electronic Data Capture; Vanderbilt University [31]) will be used for data collection and management, and auxiliary curation tools, such as REDbox [32], will ensure the quality and integrity of the collected information. In this project, we will use the REDCap instance managed by the Brazilian Society of Medical Genetics and Genomics and used by RARAS [31,33]. In addition to REDCap, the project will take advantage of the technological infrastructure of the RARAS network to implement the registry, with strict security and privacy procedures following the General Data Protection Law (GDPL) [34].

### Ethical Considerations

#### Human Participant Ethics Review Approvals

The ethics committee of the HCRP (Hospital das Clínicas da Faculdade de Medicina de Ribeirão Preto), University of São Paulo approved this project (82571424.4.0000.5440). This study will follow the guidelines of the *Good Clinical Practice Manual* of the International Conference on Harmonization [35]. Furthermore, the project will be submitted to the MoH to be included as research of strategic interest for the SUS, following Resolution 580/2018.

#### Informed Consent

The research team will obtain informed consent through the signing of an informed consent form before any patient’s involvement in the project activities. Participants will be informed of their rights, including the right to withdraw their consent at any time without any cost and request full removal from the study.

#### Privacy and Confidentiality

All research activities will be conducted accordingly to the Brazilian General Personal Data Protection Law (number 13.709/2018) and the MoH Ordinance 2073/2011. Anonymized data will only be considered personal if the anonymization and deidentification process is reversed, as defined in Article 12 of the Brazilian General Personal Data Protection Law. In addition, only nonidentifiable information may be exported or transferred, similar to analyses, reports, consolidated datasets production, or research repositories’ availability. They must not contain or use personally identifiable data, as such information is unnecessary for these activities and may violate participants’ privacy. Personal and sensitive data will be premapped, and computational techniques will be used for anonymization and pseudonymization, along with manual validation by the responsible researcher before any data are made available or transferred.

#### Compensation

No financial compensation will be provided to any participant for their involvement in this research. Nonetheless, participation is voluntary, and withdrawal will not result in any form of disadvantage or loss of health care services.

### Data Storage, Preservation, and Sharing Policies

The data will be stored on servers hosted on the cloud computing infrastructure of the University of São Paulo (USP), with a contingency physical server. Daily backups will be performed and stored on cloud storage media, separated from the sources, with a retention period of 7 days.

The data will be preserved for at least 10 years and maintained under conditions that ensure their integrity and accessibility following best data management practices. The data can be exported and made available in formats, preferably open and nonproprietary (eg, CSV).

The preservation and sharing policies aim to maximize benefits for all stakeholders while respecting ethical and privacy guidelines and encouraging reproducibility and subsequent developments. The data will be available under the Creative Commons Attribution 3.0 Unported license, which permits the data’s use, sharing, and modification, provided the source is appropriately cited. In addition, depending on the data repository, other licenses may apply. The results will also be made available through the following:

- Scientific articles to ensure research transparency and replicability
- The São Paulo State Network of Scientific Data Repositories [36,37]
- FAIRsharing [38]
- Presentations at national and international scientific events

### RD Awareness Efforts

Collaboration agreements, population screening surveys, information sharing, access to existing databases, and cooperation will be established to facilitate awareness efforts. Strategic partnerships with health care institutes, laboratories, and patient associations will also contribute to data collection, and these will collaborate with HCRP service units’ screening registry. The aim is to engage critical stakeholders in the research, creating a robust care and knowledge-sharing network. This collaboration will enhance the project’s educational aspect, yielding high-quality educational materials that contribute to RD awareness and knowledge. In addition, the specialized second opinion platform will contribute to creating a complex database based on scientific and clinical evidence derived from real problems and synchronous and asynchronous discussion groups. The knowledge produced will be public and educational, supporting HCPs’ decision-making in similar situations initially discussed through the second opinion platform.

To gather, organize, share, and disseminate structured, evidence-based knowledge, a knowledge management model will be implemented according to the context in which the HCRP operates and the demand of its audience. This model will be based on the knowledge management model for the Brazilian public administration by the Institute for Applied Economic Research (Instituto de Pesquisa Econômica Aplicada) and will focus on citizen-oriented results. This process consolidates a knowledge management plan into 4 well-defined phases: diagnosis, planning, development, and implementation [39,40]. These phases will be executed following the principles outlined in the National Health Information and Informatics Policy [41] and the information standards and technologies recommended by the World Wide Web Consortium [20].

Educational materials, including informational guides, explanatory videos, and infographics, will be available on the RARAS web portal [42]. Digital strategies, including newsletters, thematic campaigns, search engine optimization, and social media promotion, will be used to reach a broad audience. The educational intervention will target HCPs, health care students, and the general community through in-person and virtual workshops as well as partnerships with schools and patient associations, aiming to raise awareness, reduce stigma, and include people with RDs [43].

The effectiveness of interventions in network access, patient registration, and educational actions will be measured using digital strategies, pre- and postintervention questionnaires, validated assessment scales, and health outcomes evaluations. These assessments will provide a comprehensive view of changes in perception and understanding of RDs and measure the impact on network access, early diagnosis, and appropriate treatments. On the basis of the results, continuous adjustments and improvements will be made.

### Evaluation Framework

Implementation research (IR) will be conducted to explore the impact of digital technologies in real-world settings [44]. Even where there is evidence, challenges related to implementing new technology and strategy play a significant role in how proven approaches in research conditions will work in real-world situations [44].

IR represents an essential interface between the availability of tools, strategies, and interventions and their use within a health care system. The reach, effectiveness, adoption, implementation, and maintenance (RE-AIM) framework will measure the success of implementing an intervention, considering 5 essential dimensions: reach, effectiveness, adoption, implementation, and maintenance. Reach refers to the number or proportion of individuals affected by the intervention. Effectiveness refers to the intervention’s outcomes (including adverse effects, quality of life, and others) and includes reasons for the intervention’s success or the lack of positive results. Adoption refers to the proportion and representativeness of settings and providers and agents of the intervention. Implementation refers to the elements of the intervention, such as delivery on time and as intended, adaptations made, and their acceptance by end users. Maintenance measures the level at which an intervention is sustained over time, including the system, provider, and individual levels [45,46].

In addition, the Standards for Reporting Implementation Studies (StaRI) [47] will provide a comprehensive framework to enhance the transparency and consistency of reporting in IR. By distinguishing between the implementation strategy and the intervention, StaRI ensures that studies clearly describe what is being implemented and how it is delivered, facilitating replication and critical appraisal across diverse contexts.

This approach will ensure that the intervention is efficiently evaluated and optimized within the health care system. These domains are essential for the final impact of the recommendations, and each stage requires a different evaluation approach. Thus, each evaluation phase will provide valuable information and tools to support evidence-based decisions in public management [44,45]. The data analysis strategy will be developed considering different stages and types of data. For data collection at the HCRP, an initial descriptive analysis will be conducted to define disease prevalence indicators and geographic, demographic, and socioeconomic information according to the axes described in the NPCCPRD [1]. These analyses can be conducted globally, covering all HCRP units, or individually to understand the system better.

The research will not be limited to understanding patients with RDs at HCRP but will also analyze the health care system. The analysis of the collected data on services will identify relevant indicators, such as the average diagnosis time and the availability of procedures related to RDs. Experts will evaluate each indicator to define objectives and network needs. In addition, satisfaction surveys will be conducted in 2 stages: analyzing participants’ demographic information [48] and evaluating their responses using satisfaction questionnaires, aiming to identify key points to consider in notification systems and care networks [49].

The global and individual approach to measuring RDs as a public health issue helps visibility, goal identification, appropriate resource allocation, and effective interventions [13]. Therefore, analyses encompassing all proposed lines will be organized and synthesized in periodic reports, providing essential insights to support the decision-making of HCRP managers. The results will be shared with involved stakeholders, allowing continuous monitoring and adaptation of actions as needed.

### Data Analysis Methods

Following data collection, a comprehensive descriptive analysis will be performed to summarize, systematize, and present the data in an interpretable framework. This phase serves to (1) provide a rigorous overview of the processes under investigation; (2) establish foundational insights into the dataset’s structure; and (3) identify intrinsic patterns, directional trends, and statistical anomalies. Beyond understanding the data, this stage transforms raw information into actionable knowledge while ensuring transparent reporting of preliminary findings [50].

This initial analytical phase establishes the essential foundation for subsequent and more sophisticated data exploration. The descriptive insights will enable the application of advanced analytical frameworks, such as machine learning methodologies, specifically designed to extract deeper layers of knowledge from the collected data. These techniques will systematically transform raw observations into extracted knowledge by (1) revealing latent structures and nonobvious relationships within the data ecosystem, (2) quantifying complex interactions between data, and (3) generating data-driven applications. Furthermore, we aim to predict future bad outcomes based on previously observed records, thus providing relevant insights to support the project’s objectives and facilitating data-driven decision-making [51].

### Performance Evaluation Plan and Risks

Risks encompass various scientific aspects and implications for the study participants. This detailed examination is crucial for ensuring the safety and ethical integrity of the study and maintaining transparency in our research methodology. Table 1 presents the identified risks, their causes and consequences, and mitigation strategies.

**Table 1:** Risks associated with the scientific aspects related to the study and its participants.

Risks			Causes	Consequences		Mitigation measures	
**For the study**							
	Inadequate sample	Incorrect or unrepresentative selection of participants			Results not generalizable or not applicable on a large scale		Dissemination of the study to other services and competitive inclusion strategy between participating locations
	Delay in ethics approvals	Indication of pending issues by regulatory bodies			Delay in the planned date		Meetings with representatives of ethics bodies and activities review
	Difficulty in acquiring or accessing databases	Delay in the analysis process, authorization by the administrative body, and malfunction of the collection equipment or environment			Delay in the planned date for starting the analysis		Allocation of human resources
**For the participant**							
	Entry of study participants without an adequate consent process	Inadequate team training			Deviations and violations of good practices and study protocol		Training of the team and retraining, if necessary
	Errors in study procedures	Systematic errors in the details of the execution of procedures			Deviations and protocol violations, data quality issues, and bias		Team training, objective communication to those responsible, and data quality management

The performance assessment plan details the indicators used to evaluate the program’s objectives and monitor project performance. For each stage of the public management process linked to a scientific result, we present, on a scale of 0 to 4 (0=knowledge not available at the stage; 4=knowledge fully available in an appropriate time and format), the availability of scientific results, taking into account the operational aspects of the project. The relationship between the need for scientific knowledge at each stage of the public management process and its availability is presented in Table 2.

**Table 2:** Representation of the operational aspect.

Stage in public management	Scientific result	Score	Justification	Indicator
Coordination with the community and health care professionals with WG^a^ education and PCC^b^	Technical report outlining the criteria and necessary support for program formulation and scenario modeling	3	As this is an iterative and continuous evaluation strategy, some scenarios will be analyzed after the completion of the underlying steps, and a new report will be provided.	The number of people reached by educational programs and participation rate in training programs
Scenario modeling with the reference and counterreference WG	Detailed technical report with modeling results and support in defining education plans and referral criteria	4	—^c^	Number of viable scenarios and consensus between groups
Registration, monitoring, second opinion platform, and algorithms for WG implementation	Software, registration, sentinel criteria, and algorithmic association rules	2	The acquisition process and data quality may require structural adaptations and impact the scientific result.	The number of registered patients and the system use rate
Performance analysis, data quality, and processes	Performance report, data quality, and processes	4	—	Adequate referral rate and reach, adoption, effectiveness, implementation, and maintenance of actions
Regulation model	Preparation of a presentation for experts from the RARAS^d^ network and representatives of the MoH^e^	0	This is the pending stage of implementation and performance analysis of the proposed model.	Average time to access diagnostic resources
Disclosure and campaigns	Educational materials and content for campaigns, professional training, and management protocols	2	The stage is conditioned by the subsidies defined by the reference and counterreference WG and the achievement of registration implementation.	Second opinion platform use rate, the number of people reached by educational actions, and approval rate in training programs

^a^WG: working group.

^b^PCC: primary care center.

^c^Not applicable.

^d^RARAS: National Rare Diseases Network (Rede Nacional de Doenças Raras).

^e^MoH: Ministry of Health.

The scenario modeling and performance analysis, data quality, and process stages will be responsible for identifying possible needs for changes in management processes. On a scale from 0 to 4 (0=no use of scientific knowledge; 4=full use of scientific knowledge), we present the expected effects or changes in the strategic aspect of the project (Table 3).

**Table 3:** Representation of the strategic aspect.

Expected change in public management	Scientific result	Score	Justification	Indicator
Implementation of a reference and counterreferral model for RDs^a^ with screening and referral guidelines and protocols	A monitoring system with efficiency and effectiveness indicators for each implementation phase (training and registration structuring) that enables independent updating of reference scenarios by the HCRP^b^	1	The lack of financial and human resources, resistance to change on the part of HCPs^c^, and the complexity of the implementation process may not make maintaining the model sustainable.	The number of tools and guidelines available as well as the average time for diagnosis of RDs
Adoption of an effective and sustainable RD recording and monitoring system	MDS^d^, mapping between terminologies and encodings, and validated digital deployment and evaluation strategies	3	—^e^	Adhesion rate, the level of satisfaction, and HCP coverage; effectiveness and financial sustainability; and data quality and integration
Incorporation of a knowledge management model to support education and awareness processes about RDs for employees and patients	Joint action of the WG^f^ and HMs^g^ responsible for educational actions in training and disseminating digital health guidelines and strategies	2	The lack of financial resources, employee resistance, and other institutional priorities may not make maintaining the model sustainable.	Organizational knowledge management maturity level

^a^RD: rare disease.

^b^HCRP: Clinics Hospital of Ribeirão Preto (Hospital das Clínicas de Ribeirão Preto).

^c^HCP: health care professional.

^d^MDS: minimum dataset.

^e^Not applicable.

^f^WG: working group.

^g^HM: health manager.

### Expected Outcomes

#### RD Public and Scientific Management

**Figure 3:**
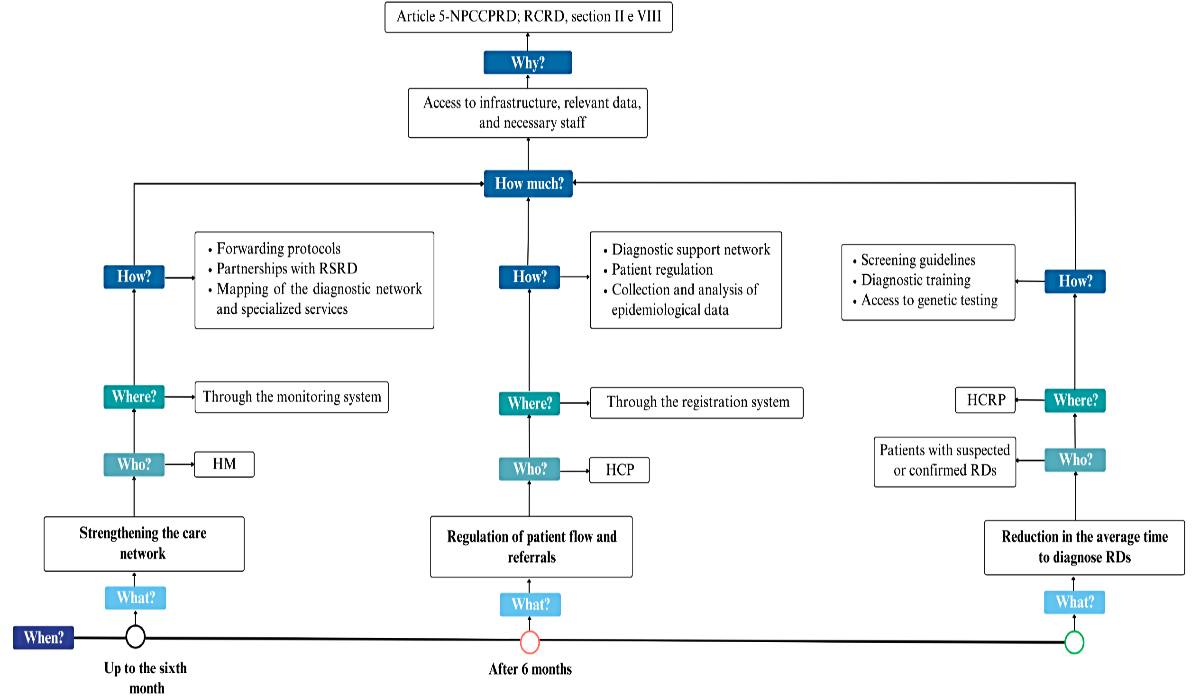
Facilitated access to the care network (line 1). HCRP: Clinics Hospital of Ribeirão Preto (Hospital das Clínicas de Ribeirão Preto); HCP: health care professional; HM: health manager; NPCCPRD: National Policy for Comprehensive Care for People with Rare Diseases; RCRD: reference center for rare diseases; RD: rare diseases; RSRD: reference service for rare diseases.

**Figure 4:**
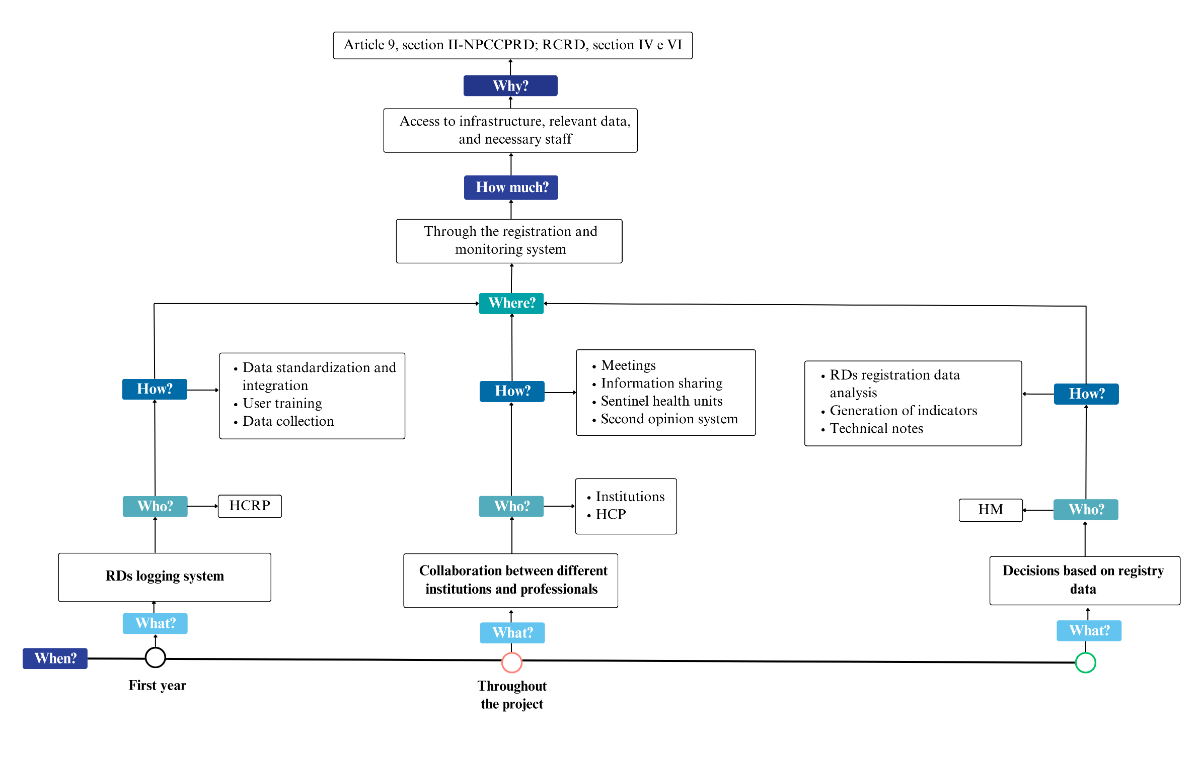
Registration and monitoring (line 2). HCRP: Clinics Hospital of Ribeirão Preto (Hospital das Clínicas de Ribeirão Preto); HCP: health care professional; HM: health manager; NPCCPRD: National Policy for Comprehensive Care for People with Rare Diseases; RCRD: reference center for rare diseases; RD: rare disease.

**Figure 5:**
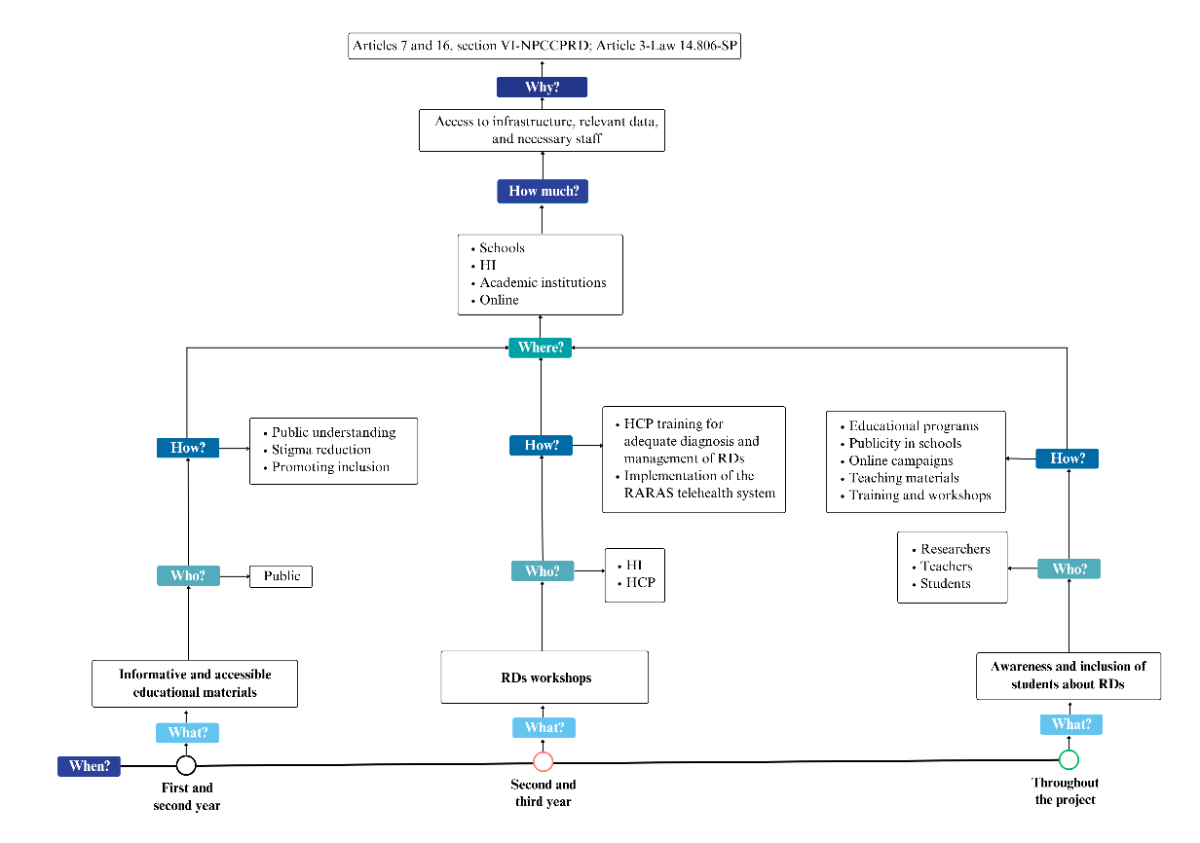
Education and awareness (line 3). HCP: health care professional; HI: health care institute; NPCCPRD: National Policy for Comprehensive Care for People with Rare Diseases; RARAS: National Rare Diseases Network (Rede Nacional de Doenças Raras); RD: rare disease.

Figures 3-5, respectively, illustrate the practical application of the 5W2H methodology for each of the project’s action lines, providing a clear overview of the planned activities, responsibilities, and required resources.

Furthermore, the project encompasses technical-scientific and innovative results through the development of patient registration and monitoring software for RDs as well as another for second opinion formation. Accordingly, it is planned to apply the intellectual property law and protect the software through registration at the National Institute of Industrial Property, allowing for technology transfer to the HCRP and, consequently, reproducibility for the SUS. This process will ensure that research conducted with public resources can be translated into societal benefits, contributing to the resolution of public health issues. In addition, detailed technical reports will facilitate the sharing of results, support the study’s replicability, and inform managers’ decision-making.

Establishing a regulatory model that emphasizes patients with RDs will include guidelines and protocols for screening and referral, which are also expected outcomes. Moreover, through the RARAS network, all results will be presented to representatives of the MoH responsible for conducting actions related to RDs in the country.

#### Produced Data and Metadata

The data and metadata produced may be structured or unstructured. Detailed clinical patient information will be collected, including diagnoses, treatments, and clinical evolution examinations. In addition, epidemiological data, such as the incidence and prevalence of RDs within the HCRP, will be recorded. For this purpose, the demographic profiles and associated risk factors of these patients will be used. When possible, registry and monitoring data will also be obtained, encompassing screenings, referrals, and outcome results from the patient’s diagnostic journey. The information generated from educational and awareness actions, including produced content, description, characterization of the target audience, and the impact of interventions, will be stored.

The metadata will complement the study set by providing (1) descriptive information related to interpreting the study results and (2) statistical information concerning the reach, effectiveness, adoption, implementation, or use of the strategies and products generated by the study’s 3 lines of action.

Furthermore, the project will use existing data from various information systems that may contain essential information about RDs. Due to the different data sources, there may be variations in formats, sizes, and structures from the initial entry of patient information to its conclusion.

The REDCap platform will ensure the secure collection and management of data. It offers a user-friendly interface for data entry, validation, and export. Each user will have password-protected access, with specific rights according to their role in the project (eg, data collector and health manager).

## Results

This project received 3 years of funding starting in February 2024. We initiated technical meetings with the IT team at the HCRP-USP and the reference management team of the State Health Department to collect data on RDs from patient medical records covering the period from January 2020 to December 2024. On August 8, 2024, we submitted the research project to the HCRP-USP Research Ethics Committee. Some missing documentation was initially identified, and we were requested to make the necessary adjustments.

After addressing these issues, the project was resubmitted to the research ethics committee on August 23, 2024, and the adjustments were approved. Once the documentation was complete, the project was forwarded for evaluation by a committee rapporteur. On November 11, 2024, the rapporteur identified additional issues, including the need for a more detailed description of the research protocols concerning the locations and health units involved, the recruitment approach and dissemination methods, the invitation to participate, explanations regarding the questionnaires, improvements to the informed consent form, and adjustments to specific fields.

The project was adjusted and revised per the suggested improvements and resubmitted to the research ethics committee. On December 9, 2024, it was approved and authorized for data collection at the study site. Following the selection and adaptation of a knowledge management maturity assessment instrument, with input from health specialists in the relevant context, on January 23, 2025, we distributed the questionnaire to health care managers involved with RDs at the study site. This step aimed to map the institution’s level of maturity in knowledge management and will guide our subsequent developments.

In parallel, we mapped the management and informational processes inherent to the domain of RDs at the study site using the business process model and notation method. On the basis of these findings, we started to structure this knowledge in a semantic and interoperable way through a computational ontology. These processes are currently under development. A scoping review is also being conducted to identify the main guidelines for education and awareness on RDs. On the basis of the preliminary results of this investigation, we developed awareness-raising materials on the topic.

These materials were used in both digital and in-person awareness actions. Among the in-person initiatives, the most notable were the activity held at the HCRP-USP during World Rare Disease Day (February 28, 2025) and the public-facing event in downtown Ribeirão Preto on March 22, 2025.

## Discussion

### Anticipated Findings

This implementation study is expected to result in 3 main contributions: improved access to comprehensive care for people with RDs through a digitalized and interoperable network; enhanced patient registration and tracking supported by standardized data structures based on findability, accessibility, interoperability, and reusability principles [52]; and strengthened awareness and knowledge dissemination through training, educational materials, and second opinion support tools. These anticipated outcomes reflect an integrated approach involving technological innovation, governance, and collaborative networks that align with national and international public health strategies for RDs.

People living with RDs often endure an arduous diagnostic journey, averaging 7 years of inconclusive consultations and potential misdiagnoses. This leads to delays in effective treatments and inefficient use of health care resources. Incorporating up-to-date scientific knowledge provides a solid foundation for informed decision-making and implementing more effective strategies. Overall, using best practices, innovative approaches, and proven solutions is essential to address patients’ challenges [53]. The engagement and use of the infrastructure and collaboration of the RARAS network, aligned with the governance and excellence of the HCRP as an RSRD, are crucial in achieving the objectives of this project. RARAS, composed of information systems [14], researchers, and specialized professionals, provides a solid foundation of collaboration and expertise needed to address the complex challenges we propose to solve. In turn, the efficient governance of the HCRP offers the necessary framework for coordinating activities, setting clear goals, and making strategic decisions.

Governance encompasses not only the internal management of the service but also network collaboration with other health care institutes, governmental bodies, and relevant stakeholders, aiming to maximize resources and optimize outcomes. At a global level, there is a consensus on the urgent need to develop a unified computerized system that allows for patient registration, monitoring, and evaluation, storing information ranging from the most basic data to screening, diagnosis, treatment, and hospitalization records. In other words, this involves digitizing the entire service to facilitate data collection and improve its analysis, visualization, and management. These tools can provide health intelligence to support evidence-based decision-making and actions, guide resource allocation and management, and assist in operationalizing the different levels of a health care network [15,54]. Despite the strong advocacy for enhanced digital technologies at national and local levels, implementing critical priorities in this research area has been slow, and actual funding remains a fraction of what is needed to make progress [55].

According to the Pan American Health Organization and the Brazilian MoH, digitalization and interoperability of health systems are fundamental to ensure more efficient and equitable care. However, implementing digital priorities at both national and local levels has been slow, delayed by inadequate funding and challenges in enhancing infrastructure [56,57]. In this context, the rapid and efficient transmission of information must be prioritized. When a manager, HCP, or patient needs to check what resources are available, they often lack the means to obtain this information. In addition, resource availability can change in real time due to the lack of interoperability between information systems, and the time spent searching for, allocating, and delivering services becomes excessive [58].

To address the prevalent problems of delayed diagnoses, inefficient resource allocation, and suboptimal patient outcomes, it is crucial to increase the availability of information and guidance for RD stakeholders in identifying appropriate institutions for specific care [14]. The interoperability of information systems is essential to ensure that there is a seamless and secure data flow among health care institutions, mitigating lengthy and inefficient processes in resource allocation. In addition, a systematic review showed that electronic health records could improve patient care by enhancing safety and lowering the risk of data errors, mainly in high-income countries, emphasizing the need for integrated systems to improve patient outcomes [59]. Studies, including one focused on COVID-19, demonstrate how frameworks, such as Hyperledger Fabric, can improve data security and interoperability, creating a patient-centric system for health data management [60].

Integrating digital technologies with international frameworks represents a pivotal innovation in RD management. It promotes interoperability and minimizes data redundancy. As Zanello et al [61] highlighted, global interoperability among clinical research networks is essential for advancing the diagnosis and treatment of RD. This project enhances data collection and analysis by adopting FAIR principles, facilitating evidence-based and timely decision-making. Implementing systems that standardize health data and enable secure sharing among stakeholders will significantly improve the quality and efficiency of care [61].

However, several limitations must be acknowledged. In regions with limited infrastructure, implementing digital tools faces significant challenges, including connectivity issues and cultural resistance to change [55]. Furthermore, the standardization of health data remains a challenge due to the diversity of systems and terminologies, underscoring the need for continued efforts toward harmonization [55]. The diversity of approaches found in the literature highlights the importance of this study in digital health as a means to advance the generation of knowledge that encompasses the complexity of using data information systems to assist in the care and follow-up of patients with RDs [14,15,62]. Through monitoring and coordination among different levels of care, information sharing through formal and informal mechanisms among various health services should acknowledge the need to use this information to guide epidemiological, clinical, and health management decision-making systems. To mitigate these limitations, the project includes codeveloped training programs, partnerships with local institutions, expert validation of ontologies and variables, and triangulation of multiple data sources to ensure data integrity.

The anticipated impact on health care systems is substantial, particularly in RD management. When integrated into digital health networks, structured data enhance operational efficiency and reduce the cognitive load on HCPs by centralizing critical information on inaccessible platforms [61].

These inefficiencies cause significant delays and low-quality service delivery despite the efforts of all stakeholders. Therefore, more access to specialized care and essential resources is needed. To address the prevalent problems of delayed diagnoses, inefficient resource allocation, and suboptimal patient outcomes, it is crucial to increase the availability of information and guidance for RD stakeholders in identifying appropriate institutions for specific care [14]. In addition, international benchmarks, such as the european reference networks, demonstrate that collaboration and interoperability can accelerate research and improve treatment access, aligning with this project’s objectives [61].

Despite establishing the NPCCPRD, practical education and awareness efforts remain challenging due to persistent stigmas and limited information about specific conditions. These problems hinder referral, diagnosis, and treatment processes, imposing a significant burden on society. To address this bottleneck, knowledge management approaches provide effective techniques, standards, and technologies to identify critical points in these processes where targeted interventions can be implemented, aligning with the objectives of the national policy [63,64].

Interventions include providing educational materials in accessible language to the public, such as posters, guides, videos, social media posts, and mobile apps, as well as conducting training sessions and workshops to enhance the curricula of HCPs. Furthermore, comprehensive awareness campaigns targeting diverse audiences and specific events, such as International Rare Disease Day, play a critical and fundamental role. Promoting and supporting networks, patient groups, and associations for individuals and families affected by these conditions is equally important [65,66].

This study will follow the StaRI to guide reporting health interventions in real-world settings to ensure methodological transparency and reporting quality. Furthermore, the RE-AIM framework remains central to the project’s evaluation strategy. These frameworks help ensure that the intervention is adaptable, sustainable, and adequately evaluated, offering insights for future scaling and policy-making.

Therefore, our proposal will benefit synergistically from the appropriation and application of scientific knowledge, resulting from the participation of a collaborative network with access to human and technological resources capable of supporting the effectiveness and efficiency of the expected interventions. The immersion and leadership of HCRP, in turn, transcend the activities carried out within the research network, as it is not only widely recognized for its excellence in research but also considered one of the 27 RSRDs in Brazil [25].

### Conclusions

This research protocol outlines a strategic, evidence-informed approach to enhancing the care of individuals with RDs in Brazil. By integrating digital health solutions, institutional collaboration, and a comprehensive evaluation framework, the project aims to address persistent challenges, such as delayed diagnosis, inefficient resource allocation, and fragmented data systems.

By leveraging HCRP’s leadership and the RARAS network’s collaborative strength, this initiative has the potential to promote interoperable data systems, improve patient tracking and follow-up, and foster educational and awareness efforts. Implementing a unified digital infrastructure guided by FAIR principles and reinforced by structured training and knowledge management is expected to contribute to scalable and sustainable improvements in care coordination.

Beyond technical advancements, the project seeks to strengthen governance practices and inform public health decision-making, ultimately contributing to more inclusive, timely, and adequate health care for people with RDs. The systematic application of the RE-AIM framework and adherence to StaRI guidelines support the study’s implementation and evaluation rigor.

These efforts are aligned with national and global priorities for RDs and illustrate how digital innovation, combined with strategic policy alignment and stakeholder engagement, can inform improvements in RD management within the SUS and beyond.

## Appendix

Multimedia Appendix 1 Interview guide.

Multimedia Appendix 2 Peer-Review Report (English Version) by the Technological Innovation Programs/PPPP - Public Policy Research Program, São Paulo Research Foundation (FAPESP, Brazil).

## Abbreviations

5W2H: what, who, where, when, why, how, and how much
HCP: health care professional
HCRP: Clinics Hospital of Ribeirão Preto (Hospital das Clínicas de Ribeirão Preto)
IR: implementation research
MoH: Ministry of Health
NPCCPRD: National Policy for Comprehensive Care for People with Rare Diseases
RARAS: National Rare Diseases Network (Rede Nacional de Doenças Raras)
RCRD: reference center for rare diseases
RD: rare disease
RE-AIM: Reach, Effectiveness, Adoption, Implementation, and Maintenance
REDCap: Research Electronic Data Capture
RSRD: reference service for rare diseases
StaRI: Standards for Reporting Implementation Studies
SUS: Unified Health System (Sistema Único de Saúde)
USP: University of São Paulo

## Data Availability

The datasets generated or analyzed during this study will not be made publicly available for privacy reasons but will be available from the corresponding author on reasonable request.

## References

[1] (2014). Portaria nº 199, de 30 de Janeiro de 2014 Institui a Política Nacional de Atenção Integral às Pessoas com Doenças Raras, aprova as Diretrizes para Atenção Integral às Pessoas com Doenças Raras no âmbito do Sistema Único de Saúde (SUS) e institui incentivos financeiros de custeio. Ministério da Saúde.

[2] Lopes-Júnior LC, Ferraz VE, Lima RA, Schuab SI, Pessanha RM, Luz GS, Laignier MR, Nunes KZ, Lopes AB, Grassi J, Moreira JA, Jardim FA, Leite FM, Freitas PD, Bertolini SR (2022). Health policies for rare disease patients: a scoping review. Int J Environ Res Public Health.

[3] Rare diseases. World Health Organization.

[4] Valdez R, Ouyang L, Bolen J (2016). Public health and rare diseases: oxymoron no more. Prev Chronic Dis.

[5] Schwartz IV, de Souza MV, Leivas PG, Schuler-Faccini L (2014). Clinical genetics and public policies: how should rare diseases be managed. Clin Biomed Res.

[6] Mayrides M, Ruiz de Castilla EM, Szelepski S (2020). A civil society view of rare disease public policy in six Latin American countries. Orphanet J Rare Dis.

[7] Pascarelli DBN, Pereira ÉL (2022). [Rare diseases in the Brazilian National Congress: analysis of parliamentary action]. Cad Saude Publica.

[8] (2012). Lei Nº 14.806, de 21 de junho de 2012. Assembleia Legislativa do Estado de São Paulo.

[9] (2015). Lei nº 15.669, de 12 de janeiro de 2015. Assembleia Legislativa do Estado de São Paulo.

[10] Global network for rare diseases. Rare Diseases International.

[11] Boycott KM, Rath A, Chong JX, Hartley T, Alkuraya FS, Baynam G, Brookes AJ, Brudno M, Carracedo A, den Dunnen JT, Dyke SO, Estivill X, Goldblatt J, Gonthier C, Groft SC, Gut I, Hamosh A, Hieter P, Höhn S, Hurles ME, Kaufmann P, Knoppers BM, Krischer JP, Macek M Jr, Matthijs G, Olry A, Parker S, Paschall J, Philippakis AA, Rehm HL, Robinson PN, Sham PC, Stefanov R, Taruscio D, Unni D, Vanstone MR, Zhang F, Brunner H, Bamshad MJ, Lochmüller H (2017). International cooperation to enable the diagnosis of all rare genetic diseases. Am J Hum Genet.

[12] Bernardi FA, Mello de Oliveira B, Bettiol Yamada D, Artifon M, Schmidt AM, Machado Scheibe V, Alves D, Félix TM (2023). The minimum data set for rare diseases: systematic review. J Med Internet Res.

[13] (2010). Health indicators for rare diseases: conceptual framework and development of indicators from existing sources. European Commission.

[14] Alves D, Yamada DB, Bernardi FA, Carvalho I, Filho ME, Neiva MB, Lima VC, Félix TM (2021). Mapping, infrastructure, and data analysis for the Brazilian network of rare diseases: protocol for the RARASne observational cohort study. JMIR Res Protoc.

[15] (2014). Providing health intelligence to meet local needs. World Health Organization.

[16] Gong S, Li D, Dong D (2020). How do patients and doctors perceive medical services for rare diseases differently in China? Insights from two national surveys. Int J Environ Res Public Health.

[17] Vandeborne L, van Overbeeke E, Dooms M, De Beleyr B, Huys I (2019). Information needs of physicians regarding the diagnosis of rare diseases: a questionnaire-based study in Belgium. Orphanet J Rare Dis.

[18] dos Santos Batista L, Kumada KM (2021). Análise metodológica sobre as diferentes configurações da pesquisa bibliográfica. Revista Brasileira De Iniciação Científica.

[19] Narayanasamy SK, Srinivasan K, Hu YC, Masilamani SK, Huang KY (2022). A contemporary review on utilizing semantic web technologies in healthcare, virtual communities, and ontology-based information processing systems. Electronics.

[20] Web standards. World Wide Web Consortium.

[21] de Lima IB, Yamada DB, Yoshiura VT, Lance RC, Lourençön Rodrigues LM, Teixeira Vinci AL, Martinho R, de Pádua SD, Charters Lopes Rijo RP, Ferreira Furegato AR, Alves D (2018). Proposal for selection of mental health indicators in the management of health networks: from heuristic to process modeling. Procedia Comput Sci.

[22] Pufahl L, Zerbato F, Weber B, Weber I (2022). BPMN in healthcare: challenges and best practices. Inf Syst.

[23] Slack N, Chambers S, Johnston R (2009). Operations Management.

[24] Caretta Teixeira JC, Bernardi FA, Lopes Rijo RP, Alves D (2021). Proposal for a health information management model based on Lean thinking. Procedia Comput Sci.

[25] (2019). Portaria nº 3.166, de 3 de Dezembro de 2019. Ministério da Saúde.

[26] Doenças raras: conhecer, acolher e cuidar. Ministério da Saúde.

[27] Lima IB, Bernadi FA, Yamada DB, Vinci AL, Rijo RP, Alves D, Furegato AR (2021). The use of indicators for the management of mental health services. Rev Lat Am Enfermagem.

[28] Bernardi FA, Alves D, Neiva MB, Yamada DB, Lima VC, Vinci A, Thomazini G, Rijo R, Felix TM (2023). A proposal for a set of attributes relevant for web portal data quality: the Brazilian Rare Disease Network case. Procedia Comput Sci.

[29] Vasant D, Chanas L, Malone J, Hanauer M, Olry A, Jupp S, Robinson PN, Parkinson H, Rath A (2014). ORDO: an ontology connecting rare disease, epidemiology and genetic data. Proceedings of the 22nd Annual International Conference on Intelligent Systems for Molecular Biology.

[30] (2019). International statistical classification of diseases and related health problems 10th revision. World Health Organization.

[31] Harris PA, Taylor R, Thielke R, Payne J, Gonzalez N, Conde JG (2009). Research electronic data capture (REDCap)--a metadata-driven methodology and workflow process for providing translational research informatics support. J Biomed Inform.

[32] Lima VC, Rijo RP, Bernardi FA, Filho ME, Barbosa-Junior F, Pellison FC, Galliez RM, Kritski AL, Alves D (2023). REDbox: a comprehensive semantic framework for data collection and management in tuberculosis research. Sci Rep.

[33] Raras. REDCap.

[34] (2018). Lei nº 13.709, de 14 de Agosto de 2018 Lei Geral de Proteção de Dados Pessoais. Presidência da República Brasília.

[35] (2005). Boas práticas clínicas: documento das Américas. Organização Pan-Americana da Saúde.

[36] Mello LE, Suman A, Medeiros CB, Prado CA, Rizzatti EG, Nunes FL, Barnabé GF, Ferreira JE, Sá J, Reis LF, Rizzo LV, Sarno L, de Lamonica R, Maciel RM, Cesar RM Jr, Carvalho R (2020). Opening Brazilian COVID-19 patient data to support world research on pandemics. Zenodo.

[37] Research data metasearch. FAPESP.

[38] Sansone SA, McQuilton P, Rocca-Serra P, Gonzalez-Beltran A, Izzo M, Lister AL, Thurston M (2019). FAIRsharing as a community approach to standards, repositories and policies. Nat Biotechnol.

[39] (2012). Modelo de gestão do conhecimento para a administração pública Brasileira. Instituto de Pesquisa Econômica Aplicada.

[40] Observatório de Gestão do Conhecimento. Instituto de Pesquisa Econômica Aplicada.

[41] (2021). Resolução nº 659, de 26 de Julho de 2021 dispõe sobre a política nacional de informação e informática em Saúde. Ministério da Saúde.

[42] RARAS - Rede Nacional de Doenças Raras homepage. RARAS.

[43] Baumann LC, Karel A, Gellman MD, Turner JR (2013). Health education. Encyclopedia of Behavioral Medicine.

[44] (2019). Recommendations on digital interventions for health system strengthening. World Health Organization.

[45] Glasgow RE, Harden SM, Gaglio B, Rabin B, Smith ML, Porter GC, Ory MG, Estabrooks PA (2019). RE-AIM planning and evaluation framework: adapting to new science and practice with a 20-year review. Front Public Health.

[46] Glasgow RE, Vogt TM, Boles SM (1999). Evaluating the public health impact of health promotion interventions: the RE-AIM framework. Am J Public Health.

[47] Pinnock H, Barwick M, Carpenter CR, Eldridge S, Grandes G, Griffiths CJ, Rycroft-Malone J, Meissner P, Murray E, Patel A, Sheikh A, Taylor SJ (2017). Standards for reporting implementation studies (StaRI) statement. BMJ.

[48] Ni K, Chu H, Zeng L, Li N, Zhao Y (2019). Barriers and facilitators to data quality of electronic health records used for clinical research in China: a qualitative study. BMJ Open.

[49] Boone HN Jr, Boone DA (2012). Analyzing Likert data. J Ext.

[50] Rouquayrol MZ, Gurgel M (2023). Rouquayrol - Epidemiologia e Saúde.

[51] Rothman KJ, Greenland S, Lash TL (2008). Modern Epidemiology.

[52] FAIR principles. GO FAIR.

[53] Félix TM, de Oliveira BM, Artifon M, Carvalho I, Bernardi FA, Schwartz IV, Saute JA, Ferraz VE, Acosta AX, Sorte NB, Alves D (2022). Epidemiology of rare diseases in Brazil: protocol of the Brazilian Rare Diseases Network (RARAS-BRDN). Orphanet J Rare Dis.

[54] Yoshiura VT, de Azevedo-Marques JM, Rzewuska M, Vinci AL, Sasso AM, Miyoshi NS, Furegato AR, Rijo RP, Del-Ben CM, Alves D (2017). A web-based information system for a regional public mental healthcare service network in Brazil. Int J Ment Health Syst.

[55] Wosny M, Strasser LM, Hastings J (2024). The paradoxes of digital tools in hospitals: qualitative interview study. J Med Internet Res.

[56] Dhingra D, Dabas A (2020). Global strategy on digital health. Indian Pediatr.

[57] (2021). Eight guiding principles of digital transformation of the health sector. A call to pan American action. Pan American Health Organization.

[58] Oliveira RA, Duarte CM, Pavão AL, Viacava F (2019). [Barriers in access to services in five health regions of Brazil: perceptions of policymakers and professionals in the Brazilian Unified National Health System]. Cad Saude Publica.

[59] Li E, Clarke J, Ashrafian H, Darzi A, Neves AL (2022). The impact of electronic health record interoperability on safety and quality of care in high-income countries: systematic review. J Med Internet Res.

[60] Khatri S, al-Sulbi K, Attaallah A, Ansari MT, Agrawal A, Kumar R (2023). Enhancing healthcare management during COVID-19: a patient-centric architectural framework enabled by hyperledger fabric blockchain. Information.

[61] Zanello G, Chan CH, Parker S, Julkowska D, Pearce DA (2024). Fostering the international interoperability of clinical research networks to tackle undiagnosed and under-researched rare diseases. Front Med (Lausanne).

[62] Delfini MG, Miyoshi NS, Alves D (2015). Minimum data consensus: essential information to continuing healthcare. Proceedings of the IEEE 28th International Symposium on Computer-Based Medical Systems.

[63] Darretxe L, Gaintza Z, Monzon-Gonzalez J (2017). A systematic review of research into rare diseases in the educational sphere. Educ Res Rev.

[64] Fakhar Manesh M, Pellegrini MM, Marzi G, Dabic M (2021). Knowledge management in the fourth industrial revolution: mapping the literature and scoping future avenues. IEEE Trans Eng Manag.

[65] Vila AC, Vila VD (2007). Trends of knowledge production in health education in Brazil. Rev Lat Am Enfermagem.

[66] Cismondi IA, Kohan R, Adams H, Bond M, Brown R, Cooper JD, de Hidalgo PK, Holthaus SM, Mole SE, Mugnaini J, de Ramirez AM, Pesaola F, Rautenberg G, Platt FM, Noher de Halac I (2015). Guidelines for incorporating scientific knowledge and practice on rare diseases into higher education: neuronal ceroid lipofuscinoses as a model disorder. Biochim Biophys Acta.

