# Supplementary material for: Promoting Comprehensive Care for People With Rare Diseases in a Tertiary Care Setting in Brazil: Protocol for a Mixed Methods Implementation Study
Source: JMIR Res Protoc. 2025 Aug 18;14:e68949. (PMC12402734)
Supplement: Multimedia Appendix 1 [file resprot_v14i1e68949_app1.docx]

**Interview Guide**

**1. Introduction**

Dear participant,

We invite you to take part in the research study entitled "*Promoting Comprehensive Care for People with Rare Diseases in a Tertiary Care Setting in Brazil*". This study is affiliated with the Department of Social Medicine at the Ribeirão Preto Medical School, University of São Paulo (USP).

Our aim is to contribute to the implementation and improvement of actions outlined in the Brazilian National Policy for Comprehensive Care for People with Rare Diseases, with a focus on enhancing healthcare and support, expediting diagnosis, fostering collaboration among institutions and professionals, providing up-to-date information, and combating the stigma associated with rare diseases.

Research Site:

Academic Complex of the Clinics Hospital of the Ribeirão Preto, Ribeirão Preto Medical School, University of São Paulo, Brazil (HCFMRP-USP).

This interview guide is intended to be applied to professionals who hold management positions within the healthcare network of HCFMRP-USP, as well as in units affiliated with the health complex of the municipality of Ribeirão Preto.

The purpose of this instrument is to assess the level of knowledge management maturity within these institutions, with the aim of subsequently developing a knowledge management model for the field of rare diseases that is tailored to the identified needs.

This instrument was adapted from the Knowledge Management Model for the Brazilian Public Administration, developed by the Institute for Applied Economic Research (Instituto de Pesquisa Econômica Aplicada - IPEA) [1], in order to meet the specific needs of the context under analysis.

The estimated time to complete this process is 20 minutes. All data will be treated confidentially, with full assurance of privacy and anonymity, in accordance with the Brazilian General Data Protection Law (Lei Geral de Proteção de Dados Pessoais - LGPD) [2].

**2. Topic Structure**

This data collection instrument is divided into seven distinct categories [1]. Each category represents a dimension that captures professionals’ perceptions regarding the described theme, taking into account the healthcare unit in which they work.

**2.1 Human Resources**

In this topic, the questions aim to identify and analyze the organization’s education and training programs, as well as examine how employees’ skills and competencies contribute to achieving organizational goals. It also assesses whether the organization disseminates relevant information to new employees, such as its policies, strategies, business model, and knowledge management tools. In addition, it examines practices such as mentoring, coaching, formal tutoring, competency-based management, promotion of teamwork, and mechanisms for recognizing and rewarding employees who share knowledge and work collaboratively.

**2.2 Processes**

This section assesses whether the organization defines its core competencies and designs its work systems and processes in a way that adds value to citizens and supports high institutional performance. It also examines whether the institution has a structured system for managing crises and unforeseen events, aiming to ensure continuity of operations. Finally, it evaluates whether the organization regularly reviews and improves its processes with the goal of enhancing overall organizational performance.

**2.3 Technology**

This section verifies whether the organization has an adequate information technology (IT) infrastructure to carry out its activities, and whether this infrastructure is aligned with the knowledge management strategy. It also assesses whether employees have effective access to the IT resources necessary for performing their duties. Additionally, it examines the use of tools such as institutional websites, intranet platforms, or internal portals for updating relevant information, enabling institutional communication, supporting knowledge transfer, and facilitating information sharing.

**2.4 Knowledge Management Processes**

This topic assesses whether the organization adopts systematic processes for the identification, creation, storage, and dissemination of structured knowledge. Additionally, the goal is to assess the existence of tools and best practices in knowledge management, such as knowledge maps, mechanisms for recording tacit knowledge, strategies for retaining the knowledge of experienced staff, adoption of management best practices, internal and external benchmarking, as well as actions focused on performance improvement and innovation.

**2.5 Leadership in Knowledge Management**

The objective of this topic is to verify whether the organization’s knowledge management vision and strategy are aligned with its strategic drivers, such as its mission, objectives, and core activities. It also seeks to identify whether there are formal organizational arrangements to implement knowledge management initiatives, whether adequate financial resources are allocated to these actions, and whether the organization has policies focused on knowledge protection and sharing, as well as on fostering collaborative work.

**2.6 Learning and Innovation**

This section assesses whether the organization articulates and promotes values related to learning and innovation, as well as whether it views mistakes as opportunities for learning. It also examines the existence of cross-functional teams for solving specific problems, the level of autonomy granted to employees by their hierarchical superiors, and the willingness of middle management to adopt new tools and work methods.

**2.7 Knowledge Management Results**

This section analyzes whether the organization has a history of implementing knowledge management processes, and whether it uses indicators to evaluate the impact of these initiatives on its organizational outcomes. It also assesses whether there has been improvement in results related to indicators of efficiency, quality, compliance, and social effectiveness as a result of implementing these actions.

**3. Closing**

To the research participant,

We would like to sincerely thank you for your participation in our research. Your contribution was of great importance to the development of this study and will certainly help achieve the proposed objectives, as well as enhance the quality of the results obtained, strengthening the advancement of knowledge in this field.

If you have any questions or would like to receive updates on the results of this study, please feel free to contact us.

This study is supported by the São Paulo Research Foundation (FAPESP) as part of the Public Policy Research Program (grant number 2023/10203-8).

**References**

1. Instituto de Pesquisa Econômica Aplicada (IPEA). Modelo de gestão do conhecimento para a administração pública brasileira. Brasília: IPEA; 2012. ISBN:9788578111397.

2. Brasil. Lei Geral de Proteção de Dados Pessoais (LGPD). 2018. Available from: https://www.planalto.gov.br/ccivil_03/_ato2015-2018/2018/lei/l13709.htm [accessed Jul 28, 2023].
