# Supplementary material for: Promoting Comprehensive Care for People With Rare Diseases in a Tertiary Care Setting in Brazil: Protocol for a Mixed Methods Implementation Study
Source: JMIR Res Protoc. 2025 Aug 18;14:e68949. (PMC12402734)
Supplement: Multimedia Appendix 2 [file resprot_v14i1e68949_app2.pdf]

Machine Translated by Google

FAPESP SAGe

DISPATCH VIEWING

**Process**	2023/10203-8
**Promotion Line**	Technological Innovation Programs / PPPP - Public Policy Research Program / PPPP - Call for Proposals (2023)
**Situation**	Running
**Validity**	01/02/2024 to 31/01/2027
**Recipient**	Domingos Alves
**Responsible**	Domingos Alves
**Institutional Link of the Process**	Faculty of Medicine of Ribeirão Preto/FMRP/USP
**Title**	Promoting Comprehensive Care for People with Rare Diseases: Strengthening the Network of Attention, Registration and Awareness at the Ribeirão Preto Clinical Hospital

Initial Proposal Dispatch Sheet - PPPP

Result

Granted

Dispatch Dates

**Issued on:**	05/12/2023

Consolidated Budget

**Benefits**	**Requested**		**Dispatch**	
	**Value (R$)**	**Value (US$)**	**Value (R$)**	**Value (US$)**
Capital				
Permanent Material	130,272.00	0.00	105,280.00	0.00
Costing				
Transportation Expenses	0.00	0.00	0.00	0.00
Daily	0.00	0.00	0.00	0.00
Consumable Material	10,468.72	0.00	10,468.72	0.00
Third Party Services	112,554.32	0.00	81,554.32	0.00
Technical Reserve - Complementary Benefits	48,000.00	0.00	72,000.00	0.00
Technical Reserve - Infrastructure Cost Direct from the Project	37,994.25	0.00	39,460.60	0.00
Provision for Import	0.00	0.00	0.00	0.00
Total	339,289.29	0.00	308,763.64	0.00

Stock Exchange Quotas

**Requested**				**Dispatch**			
**Modality / Level**	**Load Schedule**	**Duration (Months)**	**Amount**	**Modality / Level**	**Load Schedule**	**Duration (Months)**	**Amount**
MS		24	2	MS		24	2
PD		12	3	PD		12	3
TT-4	40	24	2	TT-4	40	24	2
TT-4A	40	24	1	TT-4A	40	24	1

Project Team

Team Members - Requested

**Name**	**Function**	**BC Requested**	**Requested Period**
Domingos Alves	Responsible Researcher	Yes	15/01/2024 - 14/01/2027
Amaury Lelis Dal Fabbro	Associate Researcher	-	15/01/2024 - 14/01/2027
Themis Maria Felix	Associate Researcher	-	15/01/2024 - 14/01/2027
Victor Evangelista de Faria Ferraz	Associate Researcher	-	15/01/2024 - 14/01/2027
Eric Torrieri	Associate Researcher	-	15/01/2024 - 14/01/2027
Mariane Barros Neiva	Scholarship holder	-	15/01/2024 - 14/01/2027
Marcio Eloi Colombo Jr.	Scholarship holder	-	15/01/2024 - 14/01/2027
Claudia Barbieri Tait Gandolfi	Collaborator	-	15/01/2024 - 14/01/2027
Maria Carolina de Oliveira Rodrigues	Collaborator	-	15/01/2024 - 14/01/2027
Maria Eulalia Lessa Valle Dallora	Collaborator	-	15/01/2024 - 14/01/2027
SILVIO CESAR SOMERA	Collaborator	-	15/01/2024 - 14/01/2027
TELMA MARIA OF FREITAS SIANSI	Collaborator	-	15/01/2024 - 14/01/2027
Tonicarlo Rodrigues Velasco	Contributor	-	15/01/2024 - 14/01/2027

Team Members - Dispatched

**Name**	**Function**	**BC Dispatched**	**Dispatched Period**
Domingos Alves	Responsible Researcher	Yes	01/02/2024 - 31/01/2027
Amaury Lelis Dal Fabbro	Associate Researcher	-	01/02/2024 - 31/01/2027
Themis Maria Felix	Associate Researcher	-	01/02/2024 - 31/01/2027
Victor Evangelista de Faria Ferraz	Associate Researcher	-	01/02/2024 - 31/01/2027
Eric Torrieri	Associate Researcher	-	01/02/2024 - 31/01/2027
Mariane Barros Neiva	Scholarship holder	-	01/02/2024 - 31/01/2027
Marcio Eloi Colombo Jr.	Scholarship holder	-	01/02/2024 - 31/01/2027

Claudia Barbieri Tait Gandolfi	Collaborator	-	01/02/2024 - 31/01/2027
Maria Carolina de Oliveira Rodrigues	Collaborator	-	01/02/2024 - 31/01/2027
Maria Eulalia Lessa Valle Dallora	Collaborator	-	01/02/2024 - 31/01/2027
SILVIO CESAR SOMERA	Collaborator	-	01/02/2024 - 31/01/2027
TELMA MARIA OF FREITAS SIANSI	Collaborator	-	01/02/2024 - 31/01/2027
Tonicarlo Rodrigues Velasco Contributor		-	01/02/2024 - 31/01/2027

Execution Data

**Start Date**	01/02/2024
**Duration**	36 month(s)
**End Date**	01/31/2027
**Resource allocation area**	Health
**Scientific Report (Quantity)** 3	
**Scientific Report (Dates)**	28/02/2025<br>28/02/2026<br>28/02/2027
**Accountability (Quantity)** 3	
**Accountability (Dates)**	28/02/2025<br>28/02/2026<br>28/02/2027
**Search Category**	B/T/PP

Observations / Transcriptions / Phrases

Observations to the Responsible Party

We hereby inform you that your request for research assistance, as set out in the process referred to above, has been analyzed by FAPESP advisory board, having been approved.

We would like to inform you that some of the requested budget items may not have been approved, or approved with lower or exceptional values. Please wait for the email with instructions for confirming interest by concession.

The use of the concession must follow the Instruction Manual for the Use of Resources and Accounting Aid and Technical Reserve.

To learn more about the contents of the order, please access the SAGe System (www.fapesp.br/sage) by selecting the menu item My Processes>>Process Number and, under More Information, the Dispatch option.

Please, for any query or communication regarding this correspondence, use exclusively the services of "Talk to FAPESP" at www.fapesp.br/converse.

Yours sincerely,

Marcio de Castro Silva Jr.
Scientific Director

Additional notes:
1. A budgetary scholarship quota was granted, as detailed in the Grant Term, and a deduction of previous scholarship when the rule applies.
The selected candidate may only begin their activities on the project after their registration has been approved, which must be submitted via SAGe, according to the specific Normative Instruction for each modality, available on the website FAPESP at https://fapesp.br/bolsas.
No payments or refunds will be made for scholarship holders outside the respective SAGe process.
If a PD scholarship quota has been granted, we emphasize that the approval of the candidate's registration is subject to approval of the selection process to fill the vacancy, which must strictly meet the standards described in http://www.fapesp.br/15095, especially regarding selection and dissemination criteria in media with broad international visibility in the scientific community in the area.
If you are requesting a renewal of a budget grant, please note that, in order to make it effective, it is necessary submit, in the scholarship process at SAGe, a Change Request (SM) of the Scholarship Renewal type.
Possible renewals of the aforementioned scholarship quota must be requested in the Aid, through a change request. of the initial grant, at the time of submission of the Scientific Report. The term of the grant may not exceed the validity of the aid..

Phrases for the Responsible Person

There are no associated phrases.

Transcript of Opinion for the Responsible Party

COORDINATION'S OPINION

The opinions of the Scientific Management/Business advisory team point out possible improvements to the proposal and other points of attention. Pay special attention to the comments of the Management/Business advisor in "PART B - 1.1. Justification"; and in deficiencies highlighted in "PART B - 7.2. Deficiencies and how they can be remedied". These points of attention should be commented on or reviewed in the first project report under a specific item.

Advisory 1

GENERAL GUIDELINES FOR ANALYSIS BY THE ADVISORY BOARD

The Public Policy Research Program (PPPP) is intended to support the development of research aimed at meeting specific demands of public managers oriented towards problems (social, environmental, economic, health, education, security, well-being or of any nature related to to the collective interest) and seeks to bring the São Paulo science and technology system closer to policies public or political policies implemented by civil society organizations or private entities social interest.

The Program brings together Research Institutes, Universities and entities from the State or Federal Public Sector (ministries, federal, state or municipal secretariats, state-owned companies, city halls, ministry public, courts of auditors or justice, regulatory agencies, public foundations or those linked to the government, research institutes linked to direct administration, deputies or parliamentary fronts legislative bodies, among others) and civil society organizations (NGOs, Foundations, OSCIP, Associations or Collectives). The spectrum of eligible projects is broad, both in terms of areas of activity, as to the origin and place of its execution. A basic assumption is the formation of a partnership that ensure the co-creation of policies, aiming at the use of research results in the implementation of public policies of collective interest. More information can be obtained at:).

To analyze the proposals, we ask the advisory team to comment on the following aspects: contained in this form:

Proposal Analysis. FAPESP calls the set of seven parts to be analyzed "Proposal", composed of: 1) Analysis of the research project, 2) Framing of the proposal in the notice 3) Alignment of scientific products with the demands of public management, 4) Experience of the responsible researcher and your team, 5) Merit of the scholarships requested, 6) Budget and 7) General analysis of the Proposal.

Machine Translated by Google

PART B (Public Management Consulting)

PART B - It is recommended that item 7) General Analysis of the Proposal be done after the individual analysis of the parts, refer to the most relevant parts of the previous items and, eventually, bring additional information.

PART B - 1. ANALYSIS OF THE PUBLIC MANAGEMENT PROJECT: Assess the clarity and objectivity with which the public management project and the public policies involved were described, their relevance and results expected. Assess the description of the history of public policies, the opportunity and risks of changes predicted from the research results. The need for scientific knowledge for the planned changes are a necessary condition for the approval of the merits of the proposal and its classification in the Notice. Highlight and comment on any other aspect that you consider relevant for the analysis of these aspects. Score from 0 to 4 (0 = very deficient aspect; 4 = aspect fully covered), justify for grades from 0 to 3. (mandatory field)

[ ] 0 [ ] 1 [ ] 3[[X] 2 4

PART B - 1.1. Justification:

The research project is quite complete, covering its insertion in the theme and the history of policies public policies on Rare Diseases. As it has not yet been reviewed by an ethics committee, there may be delays in execution and may require some adjustments, as per CEP guidelines.

PART B - 2. PARTNER INSTITUTION AND ASSOCIATED INSTITUTIONS (if any): Evaluate the institutions partners and associates regarding their participation and protagonism in public policies and processes management involved. Assess whether there is a need for engagement from other institutions to achieve the suggested changes. Highlight and comment on any other aspect that you consider relevant to the analysis. of this aspect. Score from 0 to 4 (0 = very deficient aspect; 4 = aspect fully covered), justify for grades from 0 to 3 (mandatory field).

[ ] 0 [ ] 1] 2 [X] 3 [ ] 4

PART B - 2.1. Justification:

I suggest that the project management team assess the involvement of the Municipal Health Department. from Ribeirão Preto and the State Department of Health of São Paulo in the project.

PART B - 3. QUALIFICATION OF THE PROPOSAL IN THE NOTICE: The PPPP is a Program aimed at research with Public Policies, carried out collaboratively between the researcher and the public manager with clearly defined public management objectives and scientific products aligned with the flow of decisions of the Public Policies involved. Research on or inspired by Public Policies, even involving topics of interest for Public Policies, carried out on the initiative of the researcher without a clearly defined public management objective or without the joint and aligned participation of the management teams research and public management throughout all stages of the process are not financed by the PPPP in none of its notices (for details see item 1 of the Notice). Grade from 0 to 4 (0 = very good aspect) deficient; 4 = aspect fully covered), justify for grades from 0 to 3 (filling in mandatory).

[ ] 0 [ ] 1 [ ] 2 [X] 3 [ ] 4

PART B - 3.1. Justification:

The objectives and expected results include the use of the system, data and other products by health services and management. And the intention is clear to contribute to decision-making based on evidence. However, instead of presenting the results to the MS managers only at the end of the project, it would be indicated a previous alignment (also with state and municipal secretariats), so that the execution is more aligned with public management.

PART B - 4. ALIGNMENT OF THE SCIENTIFIC AND MANAGEMENT COMPONENT: PPPP projects are oriented towards solving public management problems. One way to evaluate this aspect is the alignment of the product schedule and research results with the steps of the management process public. Research results must be available at the necessary times and in the necessary formats in stages of the public management process (for details see "include file link" "Example_ResearchFlow" with suggested representation of the alignment between research and management public through flowcharts and item 1.1 of the Notice). Highlight and comment on any other aspect that consider relevant for the analysis of this aspect. Score from 0 to 4 (0 = very deficient aspect; 4 = aspect fully contemplated), justify for grades from 0 to 3. (Mandatory field)

[ ] 0 [ ] 1 [ ] 2 [ ] 3 [X] 4

PART B - 4.1. Justification:

The project sufficiently details the forecast for the alignment of scientific results with the management process public, including the provision of the software that will be developed. In this sense, once again it is shown dialogue with the municipal and state health departments and the MS (already planned) is opportune.

PART B - 5. EXPERIENCE OF THE RESPONSIBLE PUBLIC MANAGER AND HIS TEAM: assess through the curriculum summaries and other descriptions of the teams of the Partner and Associated Institutions qualification for the planned activities. In the case of the responsible public manager, evaluate his/her history of public management in the topics involved in the proposal and their ability to lead the teams involved. Highlight and comment on any other aspect that you consider relevant to the analysis of this aspect. Note from 0 to 4 (0 = very deficient aspect; 4 = aspect fully covered), justify for grades from 0 to 2 (mandatory field).

[ ] 0 [ ] 1 [ ] 3 [X] [4] 2

PART B - 5.1. Justification:

PART B - 6. MERIT OF THE SCHOLARSHIPS REQUESTED FOR THE PUBLIC MANAGER TEAM: if the proposal have requested grants for the Partner Institution or Associated teams, assess the need for grants for project execution, the quality and clarity of work plans. Highlight and comment any other aspect that you consider relevant for the analysis of this aspect. Score from 0 to 4 (0 = very deficient aspect; 4 = aspect fully covered), justify for scores from 0 to 3 (filling in mandatory).

[ ] 0 [ ] 1 [ ] 2 [ ] 3 [X] 4

PART B - 6.1. Justification:

PART B - 7. GENERAL ANALYSIS OF THE PROPOSAL: considering the six aspects analyzed separately and, eventually, additional elements that you consider relevant for the general analysis of the proposal, present its merits and deficiencies. In the case of deficiencies, indicate how they can eventually be remedied. Score from 0 to 4 (0 = very deficient aspect; 4 = aspect fully addressed) (required field).

[ ] 0 [ ] 1 [ ] 2 [X] 3 [ ] 4

PART B - 7.1. Merits:

Project built in a robust manner and contextualized with public policies. Planning of activities and the planned team is consistent with the expected objectives and results.

PART B - 7.2. Deficiencies and how they can be remedied:

Greater coordination with federal, state and municipal administrations.

PART B - 8. Final Proposal Analysis (mandatory field)

[ ] Excellent
[X] Very Good
[ ] Very good with some easily correctable deficiencies
[ ] Good
[ ] Good with disabilities
[ ] Regular
[ ] With serious deficiencies

Machine Translated by Google

Advisory 2

GENERAL GUIDELINES FOR ANALYSIS BY THE ADVISORY BOARD

The Public Policy Research Program (PPPP) aims to support the development of research aimed at meeting concrete demands of public managers oriented towards problems (social, environmental, economic, health, education, security, well-being or any nature linked to the collective interest) and seeks to bring the São Paulo science and technology system closer to public policies or policies implemented by civil society organizations or private entities of social interest.

The Program brings together Research Institutes, Universities and entities from the State or Federal Public Sector (ministries, federal, state or municipal secretariats, state-owned companies, city halls, public prosecutors, audit or justice courts, regulatory agencies, public foundations or foundations linked to the government, research institutes linked to direct administration, deputies or legislative parliamentary fronts, among others) and civil society organizations (NGOs, Foundations, OSCIPs, Associations or Collectives). The spectrum of eligible projects is broad, both in terms of areas of activity and the origin and place of their implementation. A basic premise is the formation of a partnership that ensures the co-creation of policies, aiming at the use of research results in the implementation of public policies of collective interest. More information can be obtained at:).

In order to analyze the proposals, we ask the advisory team to comment on the following aspects contained in this form:

Proposal Analysis. FAPESP calls the set of seven parts to be analyzed a "Proposal", consisting of: 1) Analysis of the research project, 2) Classification of the proposal in the call for proposals, 3) Alignment of the scientific products with the demands of public management, 4) Experience of the principal investigator and his/her team, 5) Merit of the scholarships requested, 6) Budget and 7) General analysis of the Proposal.

PART A (Scientific Advisory)

PART A - It is recommended that item 7) General Analysis of the Proposal be done after the individual analysis of the parts, make reference to the most relevant parts of the previous items and, eventually, bring additional information.

PART A - 1. ANALYSIS OF THE SCIENTIFIC PROJECT: In this item, the proposal must be evaluated for its scientific merit, regardless of the public management process involved. Evaluate the inclusion of the proposal in the related areas of knowledge, its originality, adequacy of the methodology, feasibility of the deadlines for the preparation of the results and the scientific merit of the knowledge generated independently of its contribution to the public policies involved. One item that can assist in this evaluation is the suggested scientific publication protocol. The research results must have scientific merit regardless of their application in public management and be expected to be published in science.

Highlight and comment on any other aspect that you consider relevant for the analysis of these aspects. Score from 0 to 4 (0 = very deficient aspect; 4 = aspect fully covered), justify for scores from 0 to 3 (you must choose one score).] 0 [ ] 2 [ ] 3 [X] 4

[ ] 1 [

PART A - 1.1. Justification:

The scientific project is focused on three lines of action that constitute three subprojects: (1) facilitating access to the network care for people with rare diseases, (2) establish a comprehensive registry and monitoring of patients with rare diseases, and (3) promote education and awareness about rare diseases. The implementation of a computerized system for registering and monitoring patients with rare diseases in the participating units of the Ribeirão Preto Clinical Hospital (HCRP) complex is the common thread that unites the three subprojects.

A postdoctoral fellowship is requested for the development of each of the subprojects and it is expected that at least one scientific article will be published in a journal per line of action. The project also foresees the development of software for registering and monitoring patients with rare diseases and another software for providing a second formative opinion; both software must be registered with the National Institute of Intellectual Property (INPI), allowing the transfer of technology to the HCRP and, consequently, reproducibility for the SUS.

The three subprojects are part of the digital health area. The connection between digital health and rare diseases gives the project an innovative and original character. The proposed methodology is appropriate and the schedule is feasible.

PART A - 2. QUALIFICATION OF THE PROPOSAL IN THE NOTICE: The PPPP is a Program focused on research with Public Policies, carried out collaboratively between the researcher and the public manager with clearly defined public management objectives and scientific products aligned with the decision-making flow of the Public Policies involved. Research on or inspired by Public Policies, even involving topics of interest to Public Policies, carried out at the initiative of the researcher without a clearly defined public management objective or without the joint and aligned participation of the research and public management teams throughout all stages of the process, are not funded by the PPPP in any of its notices (for details, see item 1 of the Notice). Score from 0 to 4 (0 = very deficient aspect; 4 = aspect fully covered), justify scores from 0 to 3 (you must choose one score).] 0 [ ] 1] 2

[[[ ] 3 [X] 4

PART A - 2.1. Justification:

The project is fully in line with the proposal of the PPPP Call for Proposals from FAPESP. The aim is to develop digital health tools that contribute to epidemiological surveillance activities and monitoring of patients with rare diseases, as well as to collaborate with education and awareness among health professionals about rare diseases. Such tools should allow the registration, monitoring and follow-up of patients, storing everything from basic information to data related to treatments, exams and hospitalizations.

Thus, the project seeks to contribute to the implementation and improvement of the actions recommended by the National Policy for Comprehensive Care for People with Rare Diseases in the SUS.

PART A - 3. ALIGNMENT OF THE SCIENTIFIC AND MANAGEMENT COMPONENTS: PPPP projects are geared towards solving public management problems. One way to assess this aspect is to align the schedule of research products and results with the stages of the public management process. Research results must be available at the times and in the formats required for the stages of the public management process (for details, see "include link to the file "Exemplo_FluxoPesquisa" with a suggestion for representing the alignment between research and public management through flowcharts and item 1.1 of the Notice). Highlight and comment on any other aspect that you consider relevant for the analysis of this aspect. Score from 0 to 4 (0 = very deficient aspect; 4 = aspect fully covered), justify scores from 0 to 3 (you must choose one score).

[ ] 0 [ ] 1 [ ] 2 [ ] 3 [X] 4

PART A - 3.1. Justification:

Figure 1 of the project explains the intrinsic articulation between scientific products and public management in the area of attention to people with rare diseases.
The intended results of the three subprojects, from a public policy perspective, are presented in tables 1, 2 and 3 of the project.

PART A - 4. EXPERIENCE OF THE RESPONSIBLE RESEARCHER AND HIS/HER TEAM: assess, through the curriculum summaries and other descriptions of the teams of the Host Institution, their qualification for the planned activities. In the case of the responsible researcher, assess his/her academic record in the topics involved in the proposal and his/her ability to lead the teams involved. Highlight and comment on any other aspect that you consider relevant for the analysis of this aspect. Grade from 0 to 4 (0 = very deficient aspect; 4 = aspect fully covered), justify for grades from 0 to 3 (you must choose one grade).

[ ] 0 [ ] 1 [ ] 2 [ ] 3 [X] 4

PART A - 4.1. Justification:

The researcher in charge (Prof. Dr. Domingos Alves) has extensive experience with research in the area of digital health, including the theme of rare diseases, through the National Network of Rare Diseases (RARAS) project, funded by CNPq.

Three other researchers seem to me to be essential for the successful development of the proposal: Prof. Dr. Victor E. de Faria Ferraz, as Coordinator of the Reference Service for Rare Diseases at HCRP and also one of the main researchers of the RARAS project; Prof. Dr. Amaury Lelis Dal Fabbro, as Coordinator of the Primary Care Center of the basic network of the HCRP Complex; and Prof. Dr. Temix M. Felix, a geneticist affiliated with UFRGS and coordinator of the RARAS project.

PART A - 5. MERIT OF THE GRANTS REQUESTED BY THE RESEARCHER: if the proposal has requested

Machine Translated by Google

scholarships for the Host Institution teams by the responsible researcher, assess the need for grants for project execution, the quality and clarity of work plans. Highlight and comment any other aspect that you consider relevant for the analysis of this aspect. Score from 0 to 4 (0 = very deficient aspect; 4 = aspect fully covered), justify for scores from 0 to 3 (choose mandatory a note).

[ ] 0 [ ] 1] 2 [[ ] 3 [X] 4

PART A - 5.1. Justification:

3 postdoctoral scholarships with a duration of 12 months each, 2 master's scholarships with a duration of 24 months are requested. duration each and 3 technical training scholarships 4 with 24 months duration each. Applications are compatible with the design and work plans of all scholarships are distinct and clear.

PART A - 6. BUDGET: budget items must be analyzed in light of the real needs of the project. The suggested value for the acquisition of budget items and their adequacy to the financeable items (item 9) by the Notice must be evaluated. If the values are inadequate, suggest an appropriate value. Grade from 0 to 4 (0 = very deficient aspect; 4 = aspect fully covered), justify for grades from 0 to 3 (you must choose a grade).

[ ] 0 [ ] 1 [ ] 3 [X] 4[ ] 2

PART A - 6.1. Justification:

The budget includes IT equipment (physical server, notebooks, cloud server), equipment graphic for health education (booklets, flayers, posters), payment of English revision and publication fee scientific articles and hiring a third-party marketing company for consulting for 6 months. I consider the adequate budget.

PART A - 7. GENERAL ANALYSIS OF THE PROPOSAL: considering the six aspects analyzed separately and, eventually, additional elements that you consider relevant for the general analysis of the proposal, present its merits and deficiencies. In the case of deficiencies, indicate how they can eventually be remedied. Score from 0 to 4 (0 = very deficient aspect; 4 = aspect fully addressed) (choose mandatory a note).

[ ] 0 [ ] 1] 2 [[ ] 3 [X] 4

PART A - 7.1. Merits:

I consider the proposal to be impeccable. It has scientific and social merit and can contribute greatly to the organization of the network. health care for people with rare diseases in the interior of São Paulo, especially in the area covered by Regional Health Directorate (DRS) XIII.

PART A - 7.2. Deficiencies and how they can be remedied:

I do not identify any deficiencies.

PART A - 8. Final Proposal Analysis (mandatory field)

[X] Excellent
[ ] Very Good
] Very good with some easily correctable deficiencies
[[ ] Good
[ ] Good with disabilities
[ ] Regular
[ ] With serious deficiencies

Phrases for Grant Term

There are no associated phrases.

Detailed Budget - Summary Tables

Permanent Material - National

Item	Description	Requested			Dispatch		
		Qty	Unit Value (R$)	Value Total(R$)	Qty	Unit Value (R$)	Value Total(R$)
1	Dell PowerEdge T150 Tower Server with Intel® Xe processor...	1	12,202.00	12,202.00	1	12,202.00	12,202.00
2	Inspiron 15 3000 Notebook, 11th generation Intel® Core" i7-1165G...	8	4,498.00	35,984.00	6	4,498.00	26,988.00
3	Smart TV 65" UHD 4K Samsung 65AU7700, Crystal Processor 4K...	8	3,999.00	31,992.00	4	3,999.00	15,996.00
4	PowerEdge R660xs Rack Server, 2.5" Chassis with up to 8 H...	1	50,094.00	50,094.00	1	50,094.00	50,094.00
Total				130,272.00			105,280.00

Permanent Material - Imported

No benefits found.

Consumable Material - National

Item	Description	Requested Total Value(R$)	Dispatch Total Value(R$)
1	Booklets. 200 booklets, R$ 44.54 per unit 2 Flayers.	8,908.00	8,908.00
	5,000 economic flyers, R$ 13.42 for every 50 units...	1,342.00	1,342.00
3	Posters. 200 posters, R$ 109.36 for every 100 units	218.72	218.72
Total		10,468.72	10,468.72

Consumable Material - Imported

No benefits found.

Third Party Services - National

Item	Description	Requested			Dispatch		
		Qty	Unit Value (R$)	Value Total(R$)	Qty	Unit Value (R$)	Value Total(R$)
1	InterNuvem USP with 4vCPU + 16GB RAM + Linux + 1TB HD (value ...	36	349.40	12,578.40	36	349.40	12,578.40
2	Azure with NV6adsv5 + 6vCPU + Instance 55GB RAM + Linux + 18...	4	3,218.98	12,875.92	4	3,218.98	12,875.92
	Hiring of a third-party marketing company for consultancy...	6	2,150.00	12,900.00	6	2,150.00	12,900.00
4	Boosting digital materials (Google AdWords, value...	36	200.00	7,200.00	36	200.00	7,200.00
5	Payment of publication fees articles in international journals...	5	12,000.00	60,000.00	3	12,000.00	36,000.00
6	Translation, version and text review, with views on publication...	5	1,400.00	7,000.00	0	0.00	0.00
Total				112,554.32			81,554.32

Machine Translated by Google

Third Party Services - Imported
No benefits found.

Technical Reserve - Complementary Benefits

	Name	Paper	Value	Validity
**Beneficiaries**	Domingos Alves	Researcher Responsible	72,000.00	01/02/2024 to 01/31/2027
**Coin**	R$			
**Unit Value** (annual)	24,000.00			
**Reference Date**	05/12/2023			
**Benefit Value Complementary**	72,000.00			

Technical Reserve - Direct Project Infrastructure Cost - Requested

**Percentage for Reserve Technique (Country)**	15.00 %
**Percentage for Reserve Technique (Exterior)**	15.00 %
**FAPESP Dollar**	5.00
**Technical Reserve Value (R$)**	37,994.25
**Technical Reserve Value (US$)**	0.00

Technical Reserve - Direct Infrastructure Cost of the Project - Dispatch

**Percentage for Reserve Technique (Country)**	20.00 %
**Percentage for Reserve Technique (Exterior)**	20.00 %
**FAPESP Dollar**	5.05
**Technical Reserve Value (R$)**	39,460.60
**Technical Reserve Value (US$)**	0.00

Provision for Import - Requested

**Percentage for Provision for Import (Abroad)**	15.00 %
**Provision Value for Import (US$)**	0.00

Provision for Import - Dispatch

**Percentage for Provision for Import**	15.00 %
**Provision Value for Import (US$)**	0.00

Detailed Budget - Expense Items

Permanent Material - National

**Origin**	Brazil
**Amount**	1
**Description**	PowerEdge R660xs Rack Server, 2.5" Chassis with up to 8 Hard Drives (SAS/SATA), 1 CPU, PERC 11 + Intel® Xeon® Silver 4410Y 2G, 12C/24T, 16GT/s, 30M Cache, Turbo, HT (150W) DDR5-4000 + 64GB RDIMM, 4800MT/s Dual Rank + C3, RAID 1 for 2 HDDs or SSDs (Matching Type/Speed/Capacity) + Front PERC H355 Front Load + 480GB SSD SATA Read Intensive 6Gbps 512 2.5in Hot-plug AG Drive, 1 DWPD +960GB SATA SSD Read Intensive 6Gbps 512 2.5in Hot-plug AG Drive, 1 DWPD + Dual, Hot-plug, Power Supply Fault Tolerant Redundant (1+1), 800W, Mixed Mode,NAF + NBR 14136 2P+T to C13.
**Made in Brazil**	Yes
**Currency of Origin**	R$
**Unit Value**	50,094.00
**Total Value**	50,094.00
**Justification**	Contingency physical server for mirroring data and services available on the machine used in the cloud and for accessing systems on the HCRP intranet. (To be made available to the HCRP Information and Computer Center (CIA))

Permanent Material - National

**Origin**	Brazil
**Amount**	6
**Description**	Inspiron 15 3000 Notebook, 11th Gen Intel® Core" i7-1165G7 (4-core, 16GB RAM, 1TB Cache) 12MB, up to 4.7GHz) + Windows 11 Home, Portuguese + NVIDIA® GeForce® MX350, 2GB GDDR5 + 16GB DDR4 (2x8GB) 2666MHz memory; Expandable up to 16GB (2 soDIMM slots) +512GB PCIe NVMe M.2 SSD + 15.6" Full HD (1920 x 1080) WVA
**Made in Brazil**	Yes
**Currency of Origin**	R$
**Unit Value**	4,498.00
**Total Value**	26,988.00
**Justification**	Notebooks to equip the 6 Family Health Centers (Primary Care) + Center School Health Department (Secondary Care) + Rare Diseases Reference Service HCRP, in addition to being used to test and use the Second Opinion tool Formative + training

Permanent Material - National

**Origin**	Brazil
**Amount**	4
**Description**	Smart TV 65" UHD 4K Samsung 65AU7700, Crystal 4K Processor, Screen without limits, Cable Free Look, Alexa built in, One Control.
**Made in Brazil**	Yes
**Currency of Origin**	R$
**Unit Value**	3,999.00
**Total Value**	15,996.00

Machine Translated by Google

**Justification**	Televisions to equip the 6 Family Health Centers (Primary Care) + Center School Health Department (Secondary Care) + Rare Diseases Reference Service HCRP, in addition to being used to test and use the Second Opinion tool Formative + training

Permanent Material - National

**Origin**	Brazil
**Amount**	1
**Description**	Dell PowerEdge T150 Tower Server with Intel® Xeon® E-2324G Processor (3.1 GHz, 8M Cache, 4 cores/4 threads, Turbo 65W, 3200 MT/s) + 16GB RAM + 480GB SSD + 2TB HD + optional Operating System
**Made in Brazil**	Yes
**Currency of Origin**	R$
**Unit Value**	12,202.00
**Total Value**	12,202.00
**Justification**	Contingency physical server for mirroring data and services available on the machine used in the cloud and for accessing systems on the HCRP intranet. (To be available at the HCRP Rare Disease Reference Service)

Consumable Material - National

**Origin**	Brazil
**Description**	Booklets. 200 booklets, R$ 44.54 per unit
**Unit Value**	8,908.00
**Total Value**	8,908.00
**Justification**	Physical educational material to promote and raise public awareness about the project.

Consumable Material - National

**Origin**	Brazil
**Description**	Flayers. 5,000 economical flyers, R$13.42 for every 50 units
**Unit Value**	1,342.00
**Total Value**	1,342.00
**Justification**	Physical educational material to promote and raise public awareness about the project. Leaflets, posters, booklets.

Consumable Material - National

**Origin**	Brazil
**Description**	Posters. 200 posters, R$ 109.36 for every 100 units
**Unit Value**	218.72
**Total Value**	218.72
**Justification**	Physical educational material to promote and raise public awareness about the project. Leaflets, posters, booklets.

Third Party Services - National

**Origin**	Brazil
**Amount**	3
**Description**	Payment of publication fees for articles in international journals with selection editorial policy. (estimated value)
**Unit Value**	12,000.00
**Total Value**	36,000.00
**Justification**	This has been an extremely expensive item when wanting to publish in magazines. international highlights

Third Party Services - National

**Origin**	Brazil
**Amount**	6
**Description**	Hiring an outsourced marketing company for consultancy (Monthly value: R$ 2,150.00. Total value in 6 months (months 06-12 of the 1st year): R$ 12,900.00)
**Unit Value**	2,150.00
**Total Value**	12,900.00
**Justification**	Composition of digital arts, composition of arts for physical materials, copywriting, setup of promotion and dissemination platforms.

Third Party Services - National

**Origin**	Brazil
**Amount**	4
**Description**	Azure with NV6adsv5 Instance + 6vCPU + 55GB RAM + Linux + 180GB HD + GPU 1/6X A10 (monthly value: R$ 3,218.98 / Total in 4 months of use (months 04-08 of the 3rd year): R$ 12,875.92)
**Unit Value**	3,218.98
**Total Value**	12,875.92
**Justification**	Hiring of cloud server for processing activities, analysis of data, training and testing of computational models.

Third Party Services - National

**Origin**	Brazil
**Amount**	36
**Description**	InterNuvem USP with 4vCPU + 16GB RAM + Linux + 1TB HD (price per month/ for 36 months of project)
**Unit Value**	349.40
**Total Value**	12,578.40
**Justification**	Hiring a cloud server for hosting a web data collection system data, database storage, hosting API services for export, import and sharing of data.

Third Party Services - National

**Origin**	Brazil
**Amount**	36
**Description**	Boosting digital materials (Google AdWords, monthly fee: R$200.00. Total in 36 months of project: 7,200.00)
**Unit Value**	200.00
**Total Value**	7,200.00
**Justification**	Boosting digital materials in the main search engines worldwide project.

Machine Translated by Google

Third Party Services - National

**Origin**	Brazil
**Amount**	0
**Description**	Translation, version and text review, with a view to publishing the resulting article in international journals with selective editorial policy.
**Unit Value**	0.00
**Total Value**	0.00
**Justification**	In general, an impactful journal (as intended) accepts the article and asks to do a review of the article by a specialized company.

Technical Reserve - Complementary Benefits

	Name	Paper	Value	Validity
**Beneficiaries**	Domingos Alves	Researcher Responsible	72,000.00	01/02/2024 to 01/31/2027

**Coin**	R$
**Unit Value** (annual)	24,000.00
**Reference Date**	05/12/2023
**Benefit Value Complementary**	72,000.00

Technical Reserve - Direct Infrastructure Cost of the Project

**Percentage for Reserve Technique (Country)**	20.00 %
**Percentage for Reserve Technique (Exterior)**	20.00 %
**FAPESP Dollar**	5.05
**Technical Reserve Value (R$)**	39,460.60
**Technical Reserve Value (US$)**	0.00

Provision for Import

**Percentage for Provision for Import**	15.00 %
**Provision Value for Import (US$)**	0.00
